# Analysis of Protein Folding Simulation with Moving Root Mean Square Deviation

**DOI:** 10.1021/acs.jcim.2c01444

**Published:** 2023-02-23

**Authors:** Yutaka Maruyama, Ryo Igarashi, Yoshitaka Ushiku, Ayori Mitsutake

**Affiliations:** † OMRON SINIC X Corporation, Tokyo 113-0033, Japan; ‡ Department of Physics, School of Science and Technology, Meiji University, 1-1-1 Higashi-Mita, Tama-ku, Kawasaki-shi, Kanagawa 214-8571, Japan

## Abstract

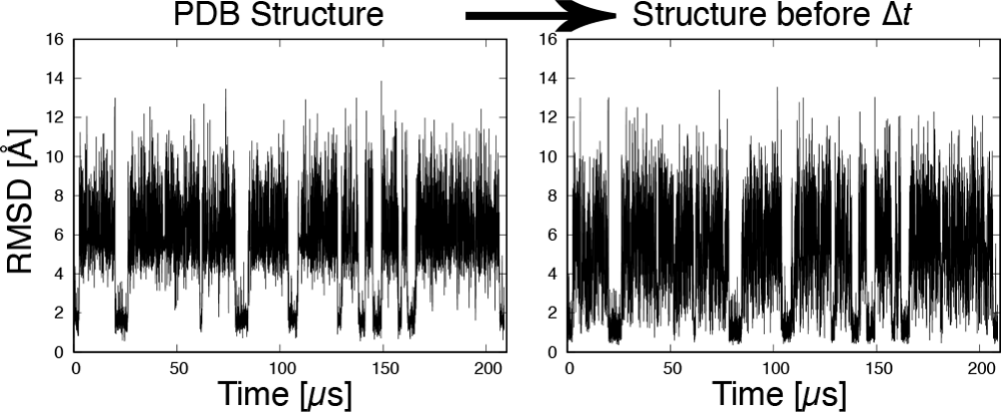

We apply moving root-mean-square deviation (mRMSD), which does not require a reference structure, as a method for analyzing protein dynamics. This method can be used to calculate the root-mean-square deviation (RMSD) of structure between two specified time points and to analyze protein dynamics behavior through time series analysis. We applied this method to the Trp-cage trajectory calculated by the Anton supercomputer and found that it shows regions of stable states as well as the conventional RMSD. In addition, we extracted a characteristic structure in which the side chains of Asp1 and Arg16 form hydrogen bonds near the most stable structure of the Trp-cage. We also determined that ≥20 ns is an appropriate time interval to investigate protein dynamics using mRMSD. Applying this method to NuG2 protein, we found that mRMSD can be used to detect regions of metastable states in addition to the stable state. This method can be applied to molecular dynamics simulations of proteins whose stable structures are unknown.

## Introduction

Molecular dynamics (MD) simulations are widely used to investigate phenomena in biomolecules such as proteins. This method is particularly suitable for investigating detailed motions at the atomic level on femto- or picosecond time scales, which are difficult to observe experimentally. In addition, application of a graphics processing unit (GPU) to scientific calculations has accelerated many MD programs, such as Amber,^1,2^ NAMD,^3,4^ OpenMM,^5^ gromacs,^6,7^ and ACEMD.^8^ Using these programs, it has become possible to perform MD simulations of the microsecond order. With the use of special purpose supercomputers for MD simulation such as MD-GRAPE^9,10^ and Anton,^11−13^ it is possible to run MD simulations on the order of milliseconds. This has made it possible to perform protein folding processes in conventional MD simulations.^14^

Long-time conventional MD simulations of protein systems are especially important when using time-dependent analysis methods, e.g., relaxation mode analysis (RMA),^15−24^ dynamic component analysis (DCA),^25,26^ and time-lagged or time-structure based independent component analysis (tICA).^27−31^ RMA was developed to investigate the dynamic properties of random spin systems^32^ and homopolymer systems for Monte Carlo^33^ and molecular dynamics.^34^ It has also been applied to biomolecular systems^15−24^ and can be used to extract slow motion that cannot be extracted by the principal component analysis because it uses time information.^15^ In free-energy topography using RMA, the minima tend to be aligned along the axis, and transitions between stable and metastable states have been well described.^16^ The differences among RMA, DCA, and tICA have been previously explained in refs (20, 21, and 23). Generating time series data to use these methods requires long-time conventional MD simulations.

In conventional MD simulation, we use a time series of root-mean-square deviation (RMSD) for heavy or C_α_ atom positions to investigate protein behavior.^35,36^ In addition, plotting various energies against RMSD is a useful approach for investigating the structural stability of a protein.^37−43^ However, the reference structure of RMSD, which, in such a case, is a native structure, must be known in advance. One possible approach would be to use an extended structure as a reference. However, in this case, the value indicating the native structure is unclear.

In the previous study, we plotted the various energies of four proteins as a function of the end-to-end distance between the N- and C-termini.^44^ The distance does not require a reference structure, and it is experimentally measurable using techniques such as fluorescent resonance energy transfer (FRET). However, the correlations between RMSD and end-to-end distance depend on individual proteins. In other words, end-to-end distance is not an appropriate indicator for investigating stable or metastable states of proteins.

In this work, we attempted to analyze the MD trajectory using time series RMSD without a reference structure. We calculated RMSD using the structure before a specified time rather than a fixed reference structure. Therefore, it is useful for MD simulations of proteins whose native structures have not yet been experimentally determined. The method of calculating such RMSD values is a known technique for checking equilibration in short-time MD simulation. This is also a simplified version of moving RMSD (mRMSD) proposed by Harada et al. for sampling efficiency of the nontargeted parallel cascade selection molecular dynamics.^45−47^ We used the time series of mRMSD to analyze Anton’s long-time simulations of the Trp-cage^48,49^ and NuG2^50^ proteins.^14^

## Methods

### Moving RMSD

RMSD is a commonly used measure for comparing protein conformations.^35,36^ We considered two different structures, v and w. These structures have *n* three-dimensional coordinates; RMSD was defined by
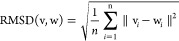
1The two protein conformations were prealigned to minimize the distance between them. There are several ways to calculate RMSD: all-atom RMSD, heavy-atom RMSD, and C_α_-atom RMSD. Here we chose C_α_-atom RMSD to investigate protein structural stability.

Conventionally, RMSD is calculated by fixing a reference. In the case of protein folding simulations, native structures obtained experimentally are used as references. Now consider a simulation of a protein whose the native structure is unknown. In the absence of a suitable reference structure, we calculate the RMSD as follows. v is a structure at time *t*, and w is one at time *t* – Δ*t*, where Δ*t* is the time interval for RMSD calculation. This description is a simplified version of the moving RMSD (mRMSD).^45,46^ In the original mRMSD, a reference is an average of several structures before and after the time *t* of interest. (See Figure 1 in ref (45).) In this work, a single structure before a certain time is used as a reference, and the RMSD between v(*t*) and w(*t* – Δ*t*) is referred to as the mRMSD. We calculated the time series of RMSD(v(*i*Δ*s*), w(*i*Δ*s* – Δ*t*)) with the sampling time interval Δ*s* at time step *i* to investigate the protein dynamics behavior. The advantage of this measure is that it does not require a reference structure. We mention that mRMSD is not suitable as an axis for energy plots because the value of mRMSD varies with time interval Δ*t*.

### Total Energy

We introduce the total energy, *E*_tot_, which is the sum of the conformational energy, *E*_conf_, and the solvation free energy (SFE), *E*_sol_:
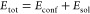
2The conformational energy is determined from the protein structure and depends on the force field used in the calculation. This energy can be calculated using molecular mechanics (MM) or MD programs. The SFE is defined as the amount of change in chemical energy when one solute molecule is moved from one position in the gas phase to another in the solvent. The SFE can be calculated using the three-dimensional reference interaction site model (3D-RISM) theory,^51,52^ the energy representation (ER) theory,^53−55^ and the grid inhomogeneous solvation theory (GIST).^56,57^ This total energy is a reasonable indicator of protein structural stability.^41−44,58^

## Computational Details

We used the following two proteins: the Trp-cage and the triple mutant of the redesigned protein G variant NuG2 (an α + β protein). The details of Anton’s MD simulations for these proteins can be found in the Supporting Information in ref (14).

We used GROMACS^59^ for calculating the conformational energy with CHARMM22* force field.^60−62^ The SFE of the proteins was calculated using the 3D-RISM theory with the reference-modified density functional theory.^63−65^ The number density of water was 0.033329 Å ^–3^ and the optimal hard sphere diameter was 2.88 Å for the thermodynamic states at 298.15 K. We performed the SFE calculation using 3D-RISM theory with the original code for GPU.^66,67^ An SFE calculation of a NuG2 protein takes 10 s on NVIDIA V100 GPU (256^3^ grids, water solvent).

Structures were extracted from Anton’s trajectory in the calculations and used every 2 ns (every 10 samples). For NuG2 protein, we used the fourth trajectory (NuG2–3 Trajectory). The total number of sampling structures was 104 400 and 86 377 for Trp-cage and NuG2, respectively. For the C_α_-RMSD calculations with respect to the native structure, the references were taken from 2JOF.pdb (model 1) and 1MI0.pdb for Trp-cage and NuG2, respectively. We used 2, 5, 10, 20, 40, and 80 ns time intervals for the mRMSD calculations. We set Δ*s* = Δ*t* in the mRMSD calculations. The end-to-end distance is defined as the distance between C_α_ atoms of the N- and C-termini. Energy calculations for Trp-cage were performed every 20 ns. In addition, calculations were performed every 2 ns during the 20–26.2 μs period.

## Results and Discussion

### Trp-cage

Trp-cage is an artificial protein consisting of 20 residues that forms a tryptophan-centered hydrophobic core. It is used as a test system for various new methods.^68−75^ In addition, Trp-cage is often used to study the folding process of proteins.^76−80^ We used the N1D/L2A/I4A triple mutant of Trp-cage^49^ in this study.

**Figure 1:**
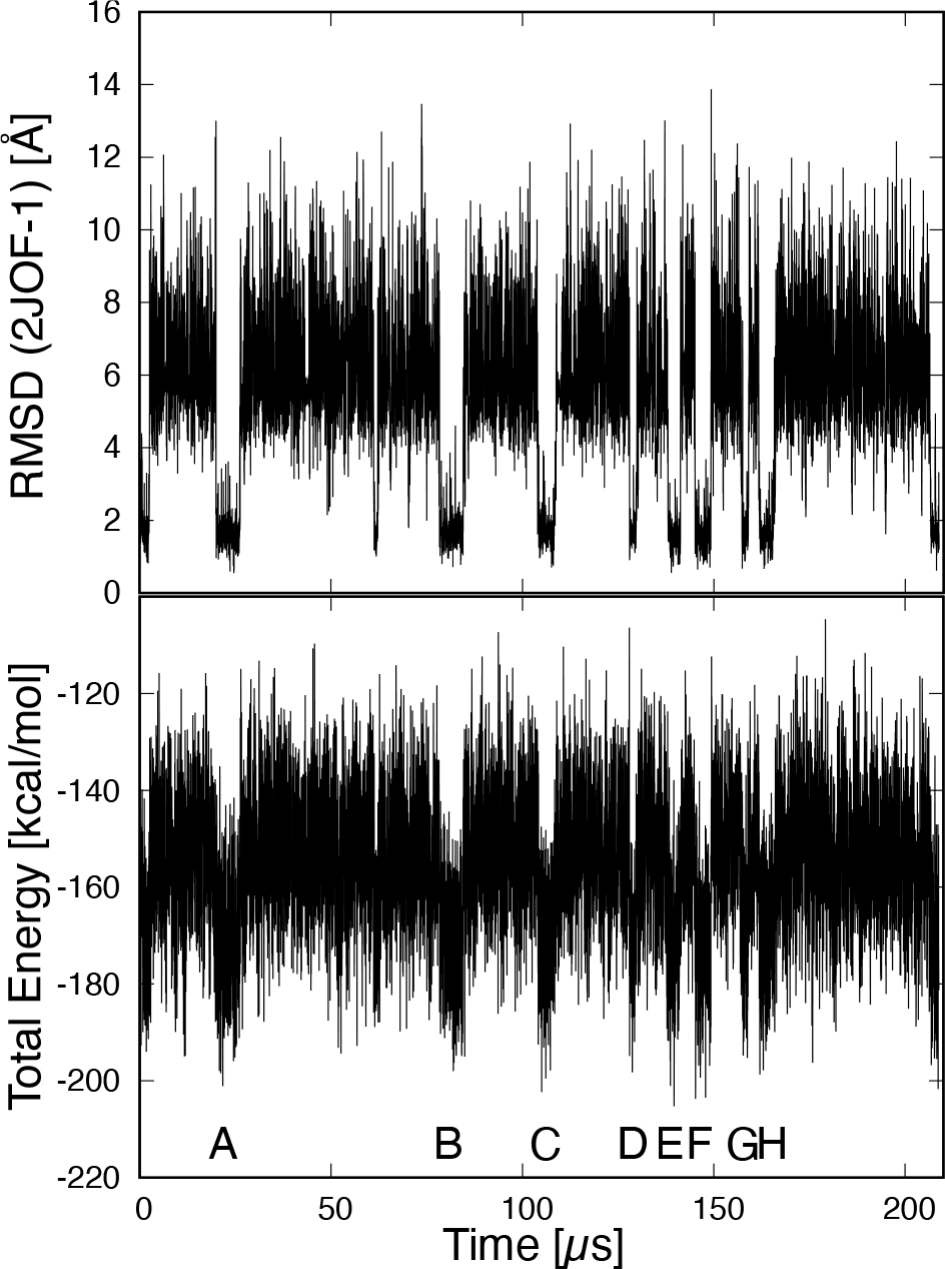
(top) Time series of the root-mean-square deviation (RMSD) with respect to the native structure of Trp-cage (PDB 2JOF, model 1). (bottom) Time series of the total energy, which is the sum of the conformational energy and the solvation free energy, of Trp-cage. A–H indicate the regions of the stable state.

The time series of the conventional RMSD of C_α_ atom from a native structure and the total energy are shown in Figure 1. Here, the native structure is the first coordinate of 2JOF.pdb. The stable state has an RMSD value of ∼2 Å, with ∼10 distinct regions. The total energy in these regions is lower than in regions with larger RMSD values. In other words, the total energy is also an indicator of the protein’s stable state. (The most stable structures in the stable state regions are shown in Figure S1 of the Supporting Information.)

**Figure 2:**
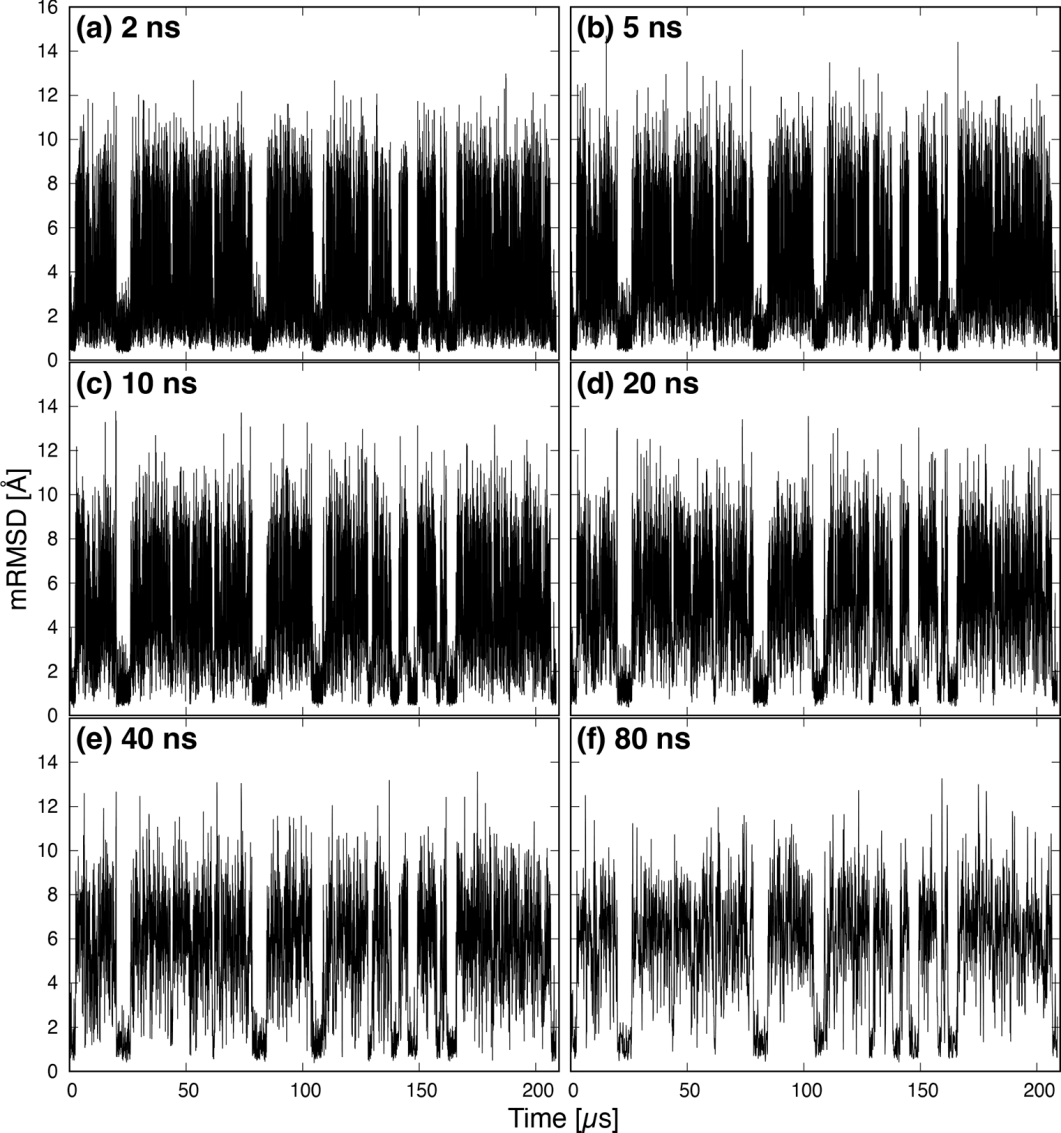
Time series of the moving root-mean-square deviation (mRMSD) of Trp-cage. Panels a–f have time intervals Δ*t* of 2, 5, 10, 20, and 80 ns, respectively.

Figure 2 shows the mRMSD time series of the C_α_ atom with time intervals as follows: (a) 2, (b) 5, (c) 10, (d) 20, (e) 40, and (f) 80 ns. At all time intervals, we were able to identify the regions of the stable state indicated by the conventional RMSD. In the stable region, the mRMSD shows values of ∼1 Å while the conventional RMSD shows values of ∼2 Å. However, in (a) 2 and (b) 5 ns, where time intervals were short, the mRMSD values were less than 2 Å even in the nonstable state. This is because the conformational change of the protein is smaller when the time interval is short. As the time interval increased to (c) 10 or (d) 20 ns, the mRMSD values in the nonstable state region increased while the values in the stable state did not change. As the time interval is further lengthened, the separation between stable and nonstable states becomes clearer (Figure 2e,f).

**Figure 3:**
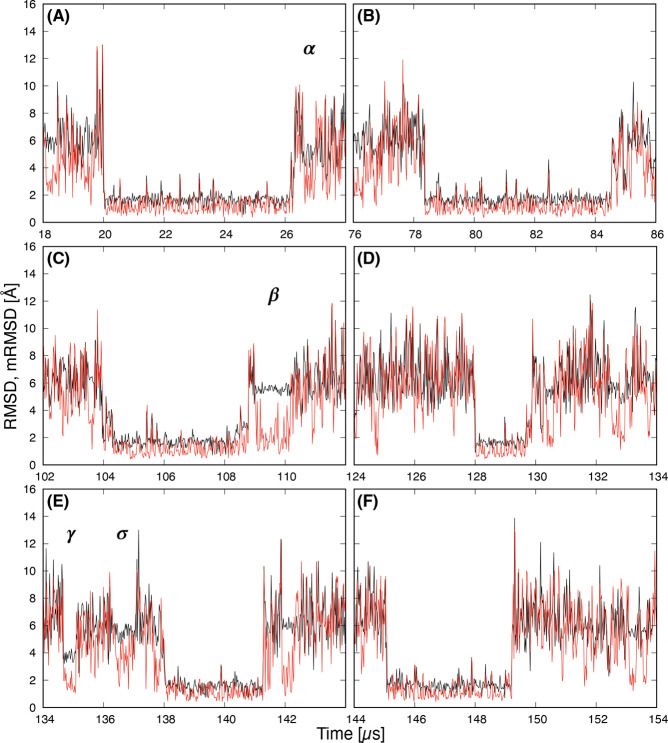
Time series of the RMSD with respect to the native structure (black) and the mRMSD with 20 ns time interval (red). Labels A–F correspond to the region of the stable state in Figure 1. α, β, γ, and σ indicate the regions of metastable states.

Next, we show the comparison of RMSD (black) and the mRMSD with a 20 ns time interval (red) near the stable state regions in Figure 3. Labels A–F correspond to A–F of the stable state regions shown in Figure 1. The mRMSD values are low and stable in all stable state regions indicated by RMSD. This suggests that the mRMSD functions properly as an indicator of the stable state. The regions indicated by α, β, γ, and σ in Figure 3 are metastable state regions. In these regions, discrepancies between RMSD and mRMSD values were observed. In addition, the fluctuations in values were small for RMSD, but large for mRMSD. This can be explained as follows. In the conventional RMSD, which uses a reference, the changes of the RMSD value become smaller when the difference between the structure and the reference is large. On the other hand, the mRMSD is more sensitive to structural changes. In the metastable state, the value of mRMSD fluctuates significantly because the structural fluctuation is larger than in the stable state.

**Figure 4:**
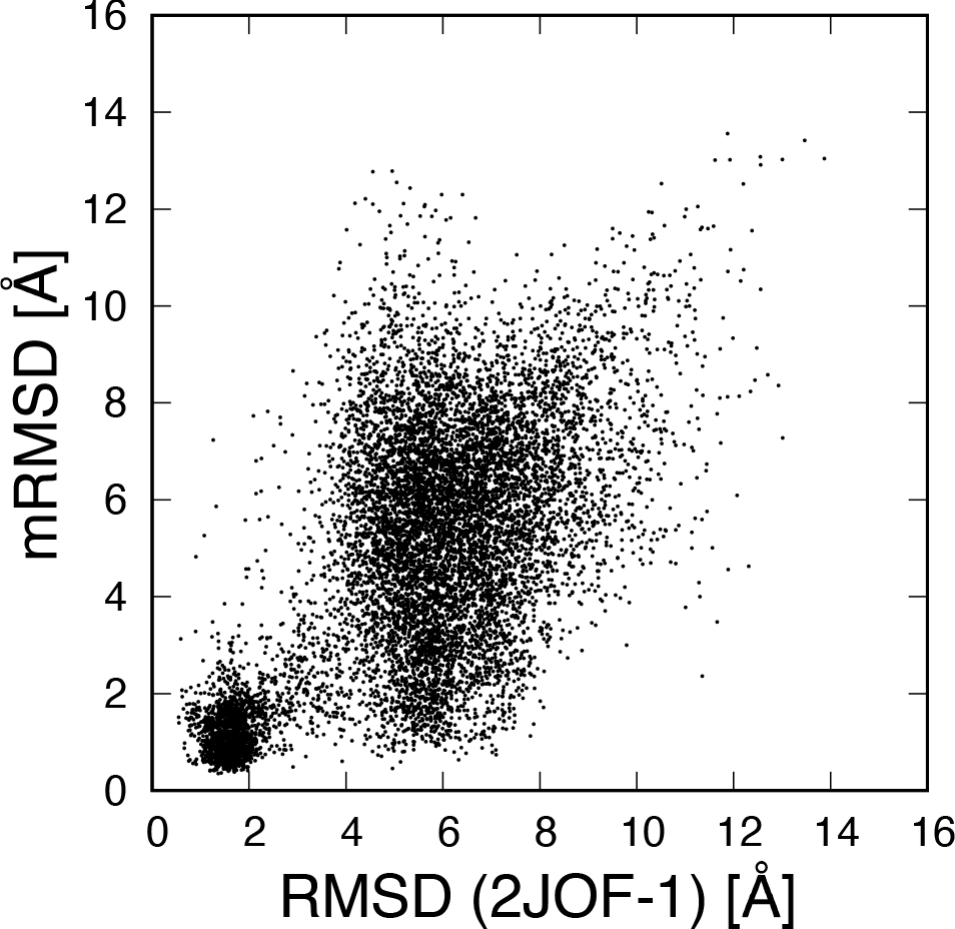
Correlation between the RMSD with respect to the native structure and the mRMSD with 20 ns time interval.

Figure 4 shows the correlation between the RMSD and the mRMSD with a 20 ns time interval. A circular region centered at point (1.5, 1.5) is observed. This indicates that there was a clear correlation in the stable state region. On the other hand, around RMSD 6 Å, the distribution extends vertically. Around RMSD 6 Å, there were various structures that deviated from the stable state. Since these structures undergo various fluctuations and structural changes, the mRMSD values fluctuate. The pattern of the correlation distribution depends on the time interval of mRMSD, but the correlation in the stable state remains independent of the time interval.

**Figure 5:**
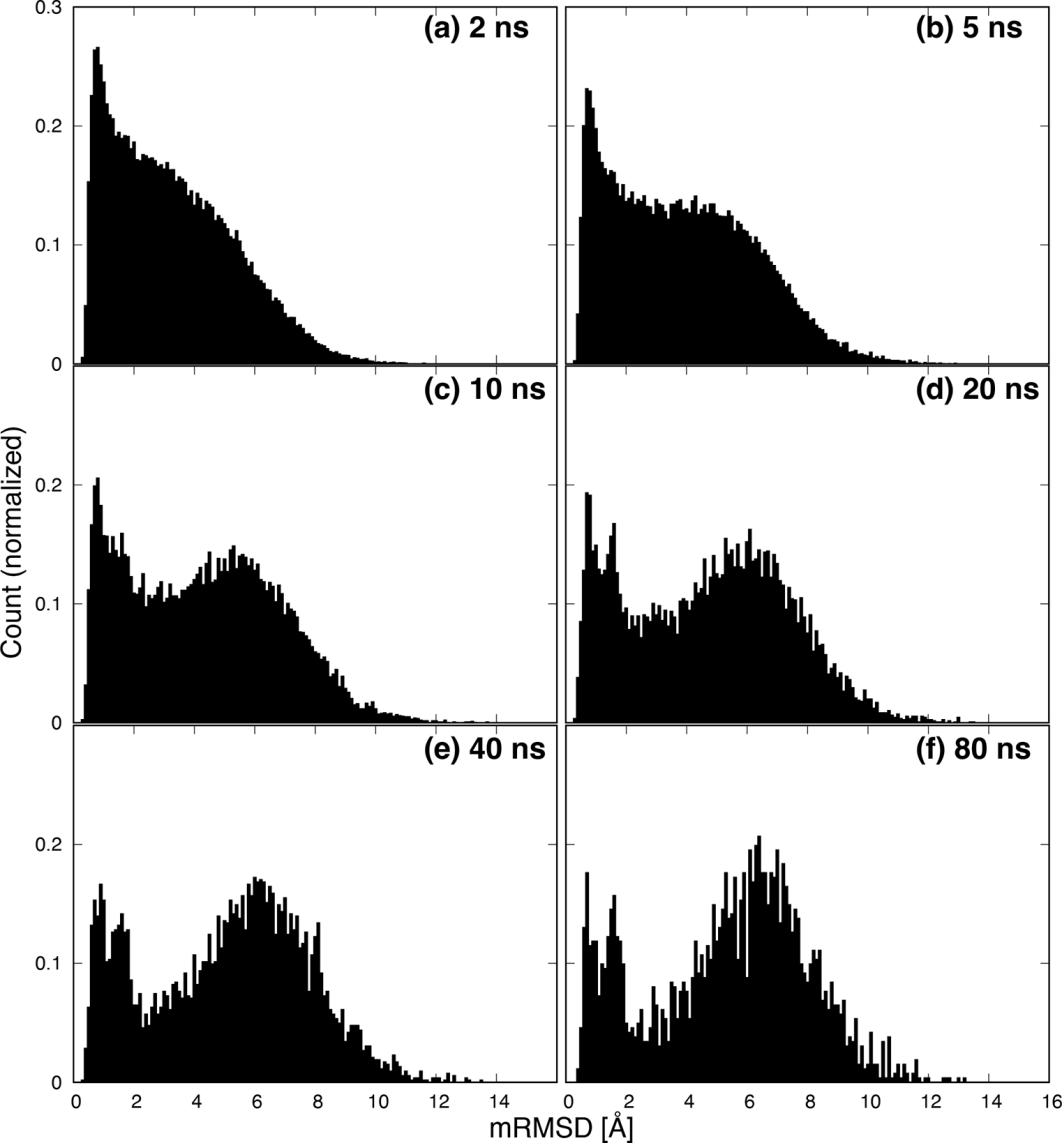
Histogram of mRMSD values. Panels a–f have time intervals Δ*t* of 2, 5, 10, 20, 40, and 80 ns, respectively. Counts are normalized.

Figure 5 shows histograms of mRMSD values. The counts of histograms are normalized. At (a) 2 ns, there is a strong peak near 1 Å that falls gently to 10 Å. However, as the time interval increases from (b) 5 ns to (c) 10 ns, a new peak becomes visible around 6 Å. This indicates that when the mRMSD time interval is short, the conformational change of the protein is too small to detect the movement of several tens of nanoseconds. Furthermore, at (d) 20 ns, the first peak splits into two peaks at 0.7 and 1.6 Å. We will discuss the peak at 1.6 Å later. Further increasing the time interval does not significantly change the position of the peak or the shape of the histogram (Figure 5e,f). Therefore, we consider that a mRMSD time interval of ≥20 ns is appropriate for analysis of protein dynamics.

**Figure 6:**
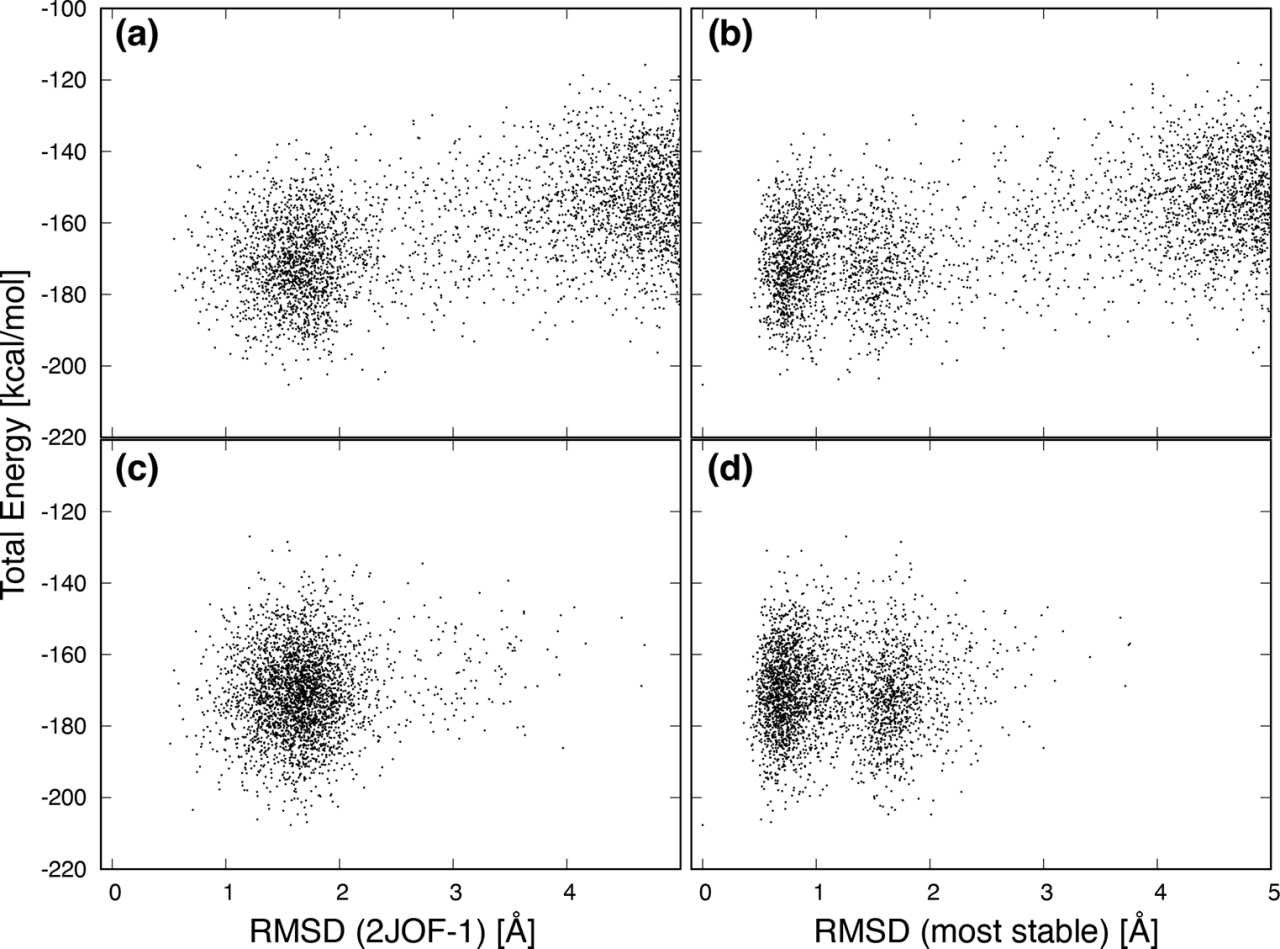
Total energy as a function of the RMSD with respect to (a, c) the native structure (PDB 2JOF, model 1) and (b, d) the most stable structure in the simulation. Panels a and b were sampled at 20 ns intervals from the entire region, while panels c and d were sampled at 2 ns intervals from the stable state region A (20.0–26.2 μs).

We show the values of the total energy of Trp-cage as functions of C_α_-RMSD in Figure 6. Panels a and b were sampled every 20 ns from the entire trajectory, while panels c and d were sampled every 2 ns in region A (20–26.2 μs) of Figure 1. In Figure 6a,c, the first structure of 2JOF.pdb was used as a reference for C_α_-RMSD, and in Figure 6b,d, the structure with the lowest total energy in each sample was used as a reference. Panels c and d assume that the MD calculation was able to sample sufficient stable structures in region A. Comparing panels a and c, the lowest total energy in Figure 6a is −205.3 kcal/mol, while it is −206.9 kcal/mol in Figure 6c. In addition, there are seven structures below −200 kcal/mol in Figure 6a, while there are 19 in Figure 6c. Naturally, it is possible to obtain lower energy structures by intensively sampling stable state regions. In other words, if the goal is to find a stable structure of a protein, stopping the MD simulation at the end of region A and sampling will achieve this. Next, comparing panel a with panel b and panel c with panel d, the separated distributions are observed around 0.8 and 1.6 Å in Figure 6b,d. These separations of distributions correspond to the separations of the first peak observed in Figure 5d,e,f. This suggests that Trp-cage has a structure with a similar shape to the stable structure. On the other hand, it is difficult to distinguish these two in Figure 6a,c. Thus, depending on the structure used as a reference, the value of RMSD will change, and the information obtained from it will vary.

**Figure 7:**
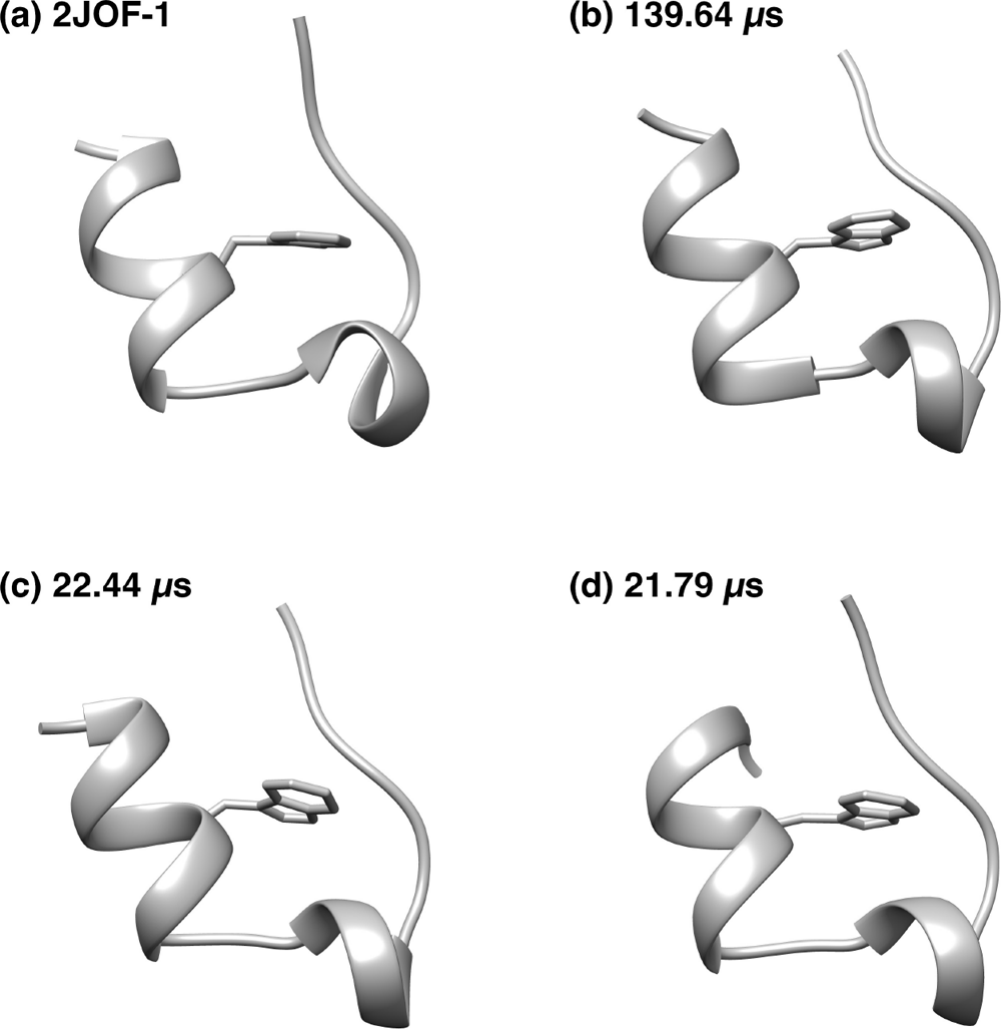
Backbone structures with the side chain of the sixth amino acid tryptophan. (a) PDB 2JOF, model 1. (b) The most stable structure when sampling the entire region at 20 ns intervals. (c) The most stable structure when sampling the stable state region A (20.0–26.2 μs) at 2 ns intervals. (d) The most stable structure with RMSD value ∼1.6 Å in Figure 6d.

Figure 7 shows Trp-cage backbone structures with the side chain of the sixth amino acid tryptophan. Panel a is the first structure of 2JOF.pdb, panels b and c are the most stable structures in Figure 6a,c, respectively, and panel d is the most stable structure, with an RMSD value ∼1.6 Å in Figure 6d. Structures a, b, and c are very similar in shape, but structure a differs in the shape of the central helix. Except for this, structures a and c are very similar in terms of the shape of the first helix. We can say that structure c is an intermediate structure between structures a and b. Structure d is close to the most stable structure, but the N-terminus is closer to the C-terminus. The N-terminal Asp1 side chain forms a hydrogen bond with the side chain of Arg16. It is well-known that the side chain of Arg16 forms a hydrogen bond with the side chain of Asp9 in the stable structure of Trp-cage.^49,81−87^ In Figure 7d, Arg16 forms hydrogen bonds with the side chains of both Asp1 and Asp9.

**Figure 8:**
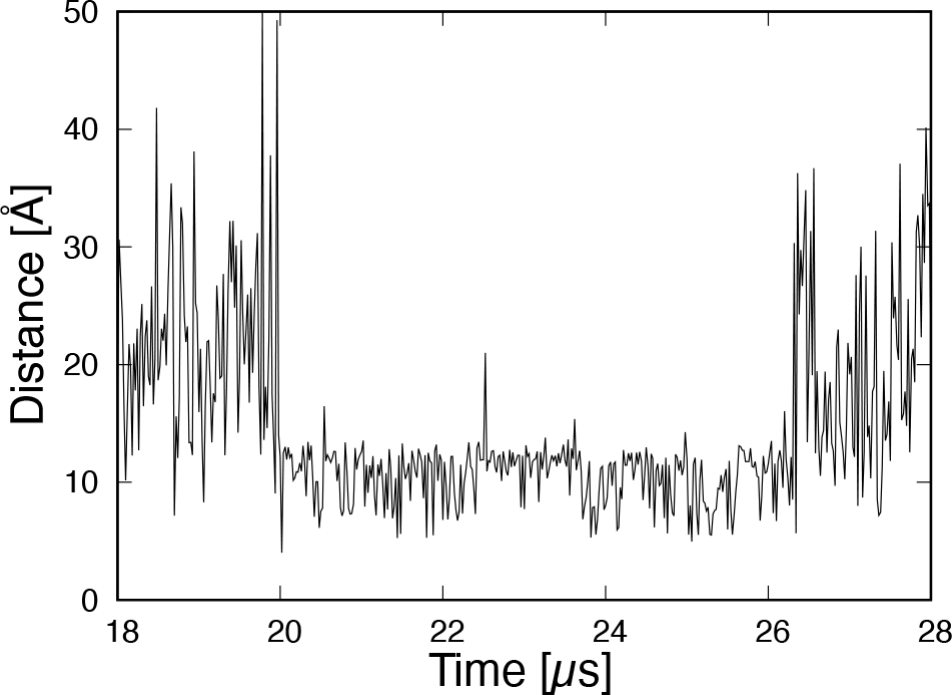
Time series of the end-to-end distance between the N- and C-termini in the stable state region A (20.0–26.2 μs).

We calculated the end-to-end distance in region A (Figure 8). Between 20 and 26.2 μs, the end-to-end distance moves back and forth between values of 8 and 12 Å. The number of oscillations is 107, giving an average period of ∼58 ns. It was found that a mRMSD time interval of ≥20 ns was required to detect this mode of hydrogen bond formation and breakage. Since this phenomenon lasts for ≥6 μs, it is not expected to have a significant effect on the structural stability of the Trp-cage.

**Figure 9:**
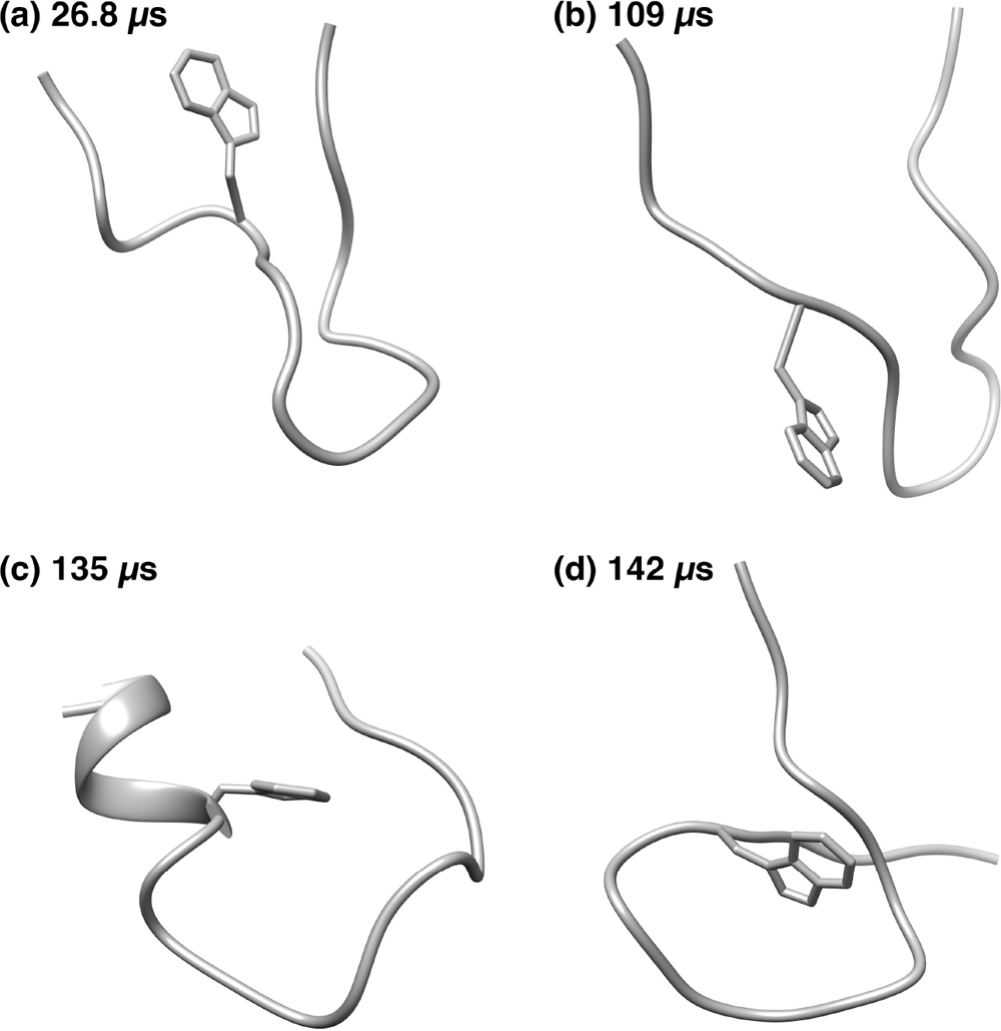
Backbone structures with the side chain of the sixth amino acid tryptophan for the metastable regions (a) α, (b) β, (c) γ, and (d) δ in Figure 3.

At the end of this subsection, we show the structures of the metastable regions in Figure 9. Panels a, b, c, and d are the structures in the metastable regions of α, β, γ, and σ in Figure 3, respectively. These structures are not exactly in a metastable state because they do not form secondary structures. They are similar to those analyzed for clustering in previous studies.^88−90^ These structures form loops and are further maintained by contact between hydrophobic side chains. By examining the time series of mRMSD, we can find states that are different from the native structure but less fragile.

### NuG2

NuG2, which contains 56 amino acids, is the N37A/A46D/D47A triple mutant of protein G.^50^ NuG2 protein consists of a single α-helix packed against two β sheets. In other words, the first β (β_1_) strand, the second β (β_2_) strand, the α-helix, the third β (β_3_) strand, and the fourth β (β_4_) strand are arranged in order from the N-terminus to the C-terminus. NuG2 is redesigned to switch folding pathways and to fold through a similar pathway to protein L in the early folding process. In the early folding process, the first and second β strands formed an N-terminal antiparallel β-sheet (β-hairpin). NuG2 has been analyzed by several groups using Anton’s MD trajectories.^41,91−98^ In addition, we have suggested the existence of many metastable states in a previous study using RMA.^22^

**Figure 10:**
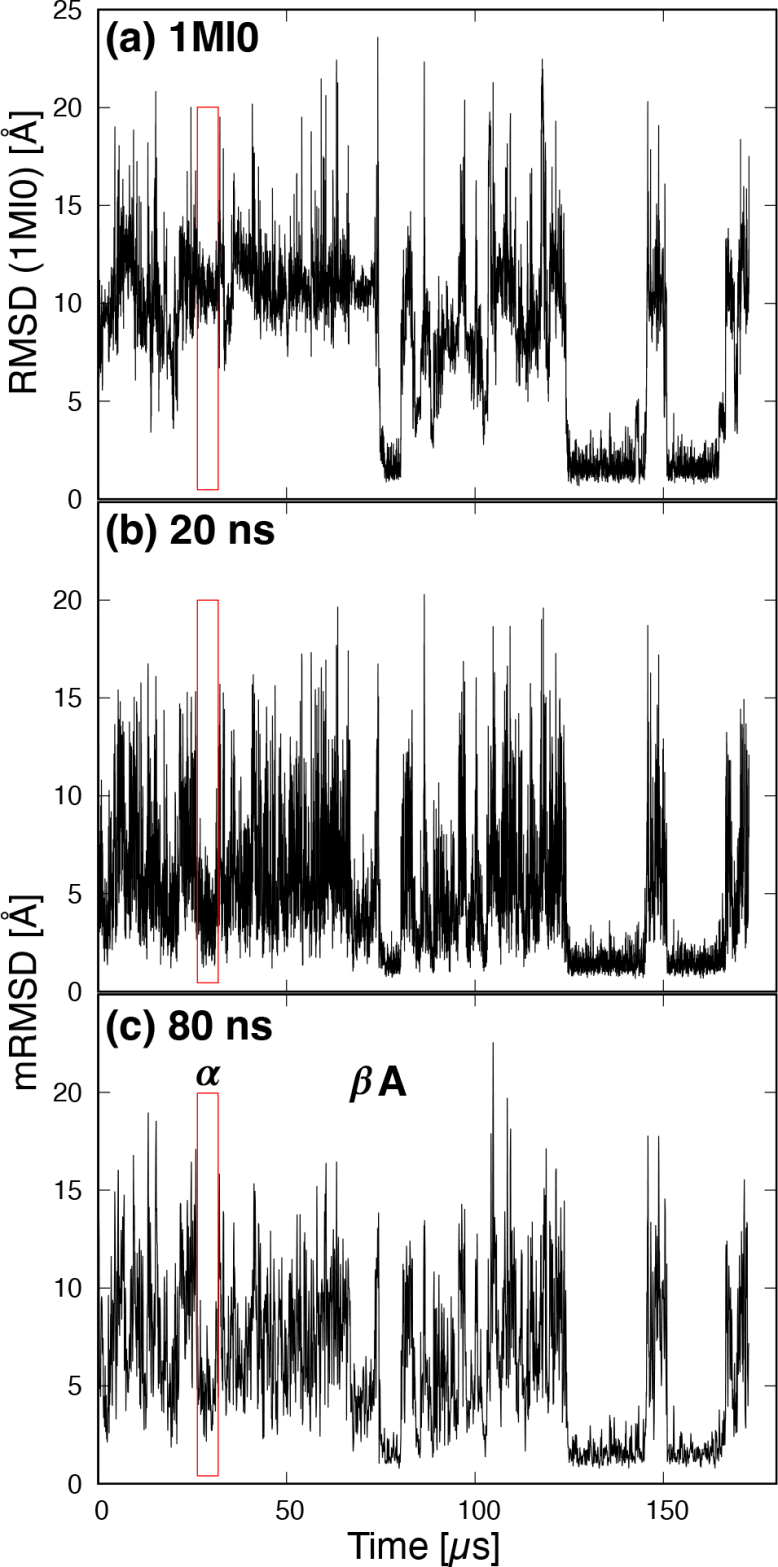
Time series of (a) the RMSD of NuG2 protein with respect to native structure (PDB 1MI0) and the mRMSD with time interval (b) 20 ns and (c) 80 ns. α and β indicate the metastable state regions, and A is the stable state region. The red rectangle indicates the extent of region α.

Figure 10 shows the time series of (a) the RMSD of the native structure (PDB 1MI0) and the mRMSD with time intervals of (b) 20 ns and (c) 80 ns. (See Supporting Information for the time series of mRMSD with time intervals 2, 5, 10, and 40 ns.) The three stable state regions with lower RMSD ∼2 Å are shown in Figure 10a. The first one is designated as stable state region A in Figure 10c. As in the case of Trp-cage, the stable state regions can be seen in the same location in Figure 10b,c. In Figure 10c, α and β indicate the metastable regions, which have slower structural changes. In both RMSD and mRMSD, the beginning and end of the region β can be clearly identified. On the other hand, it is difficult to determine the starting point of region α from the time series of RMSD. This shows the superiority of mRMSD, which can be used to calculate the difference from the structure before a certain time interval. The mRMSD is a very useful value because it can extract regions of the stable structure and metastable structures in which structural changes are slow.

**Figure 11:**
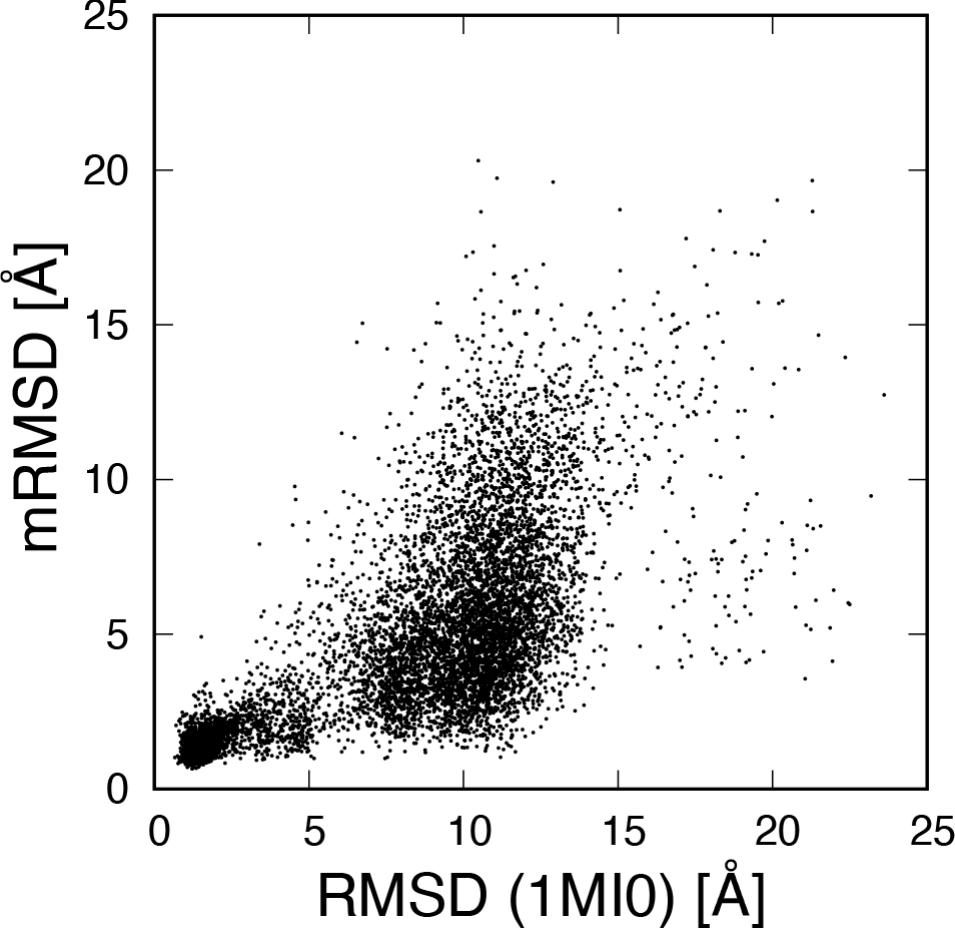
Correlation between the RMSD with respect to the native structure and the mRMSD with 20 ns time interval.

The correlation between the RMSD and the mRMSD with a 20 ns time interval is shown in Figure 11. Similar to the Trp-cage, there is a strong correlation near the region centered at (2, 2), which indicates the stable state. Compared with the Trp-cage correlation, longitudinally extending distribution is shifted around RMSD 10 Å. This is because the value of RMSD for unstable structures is protein dependent. Structures with mRMSD values smaller <5 Å are considered to be metastable states because of small structural changes. In other words, we can obtain information on metastable structures using mRMSD even if the RMSD value is large.

**Figure 12:**
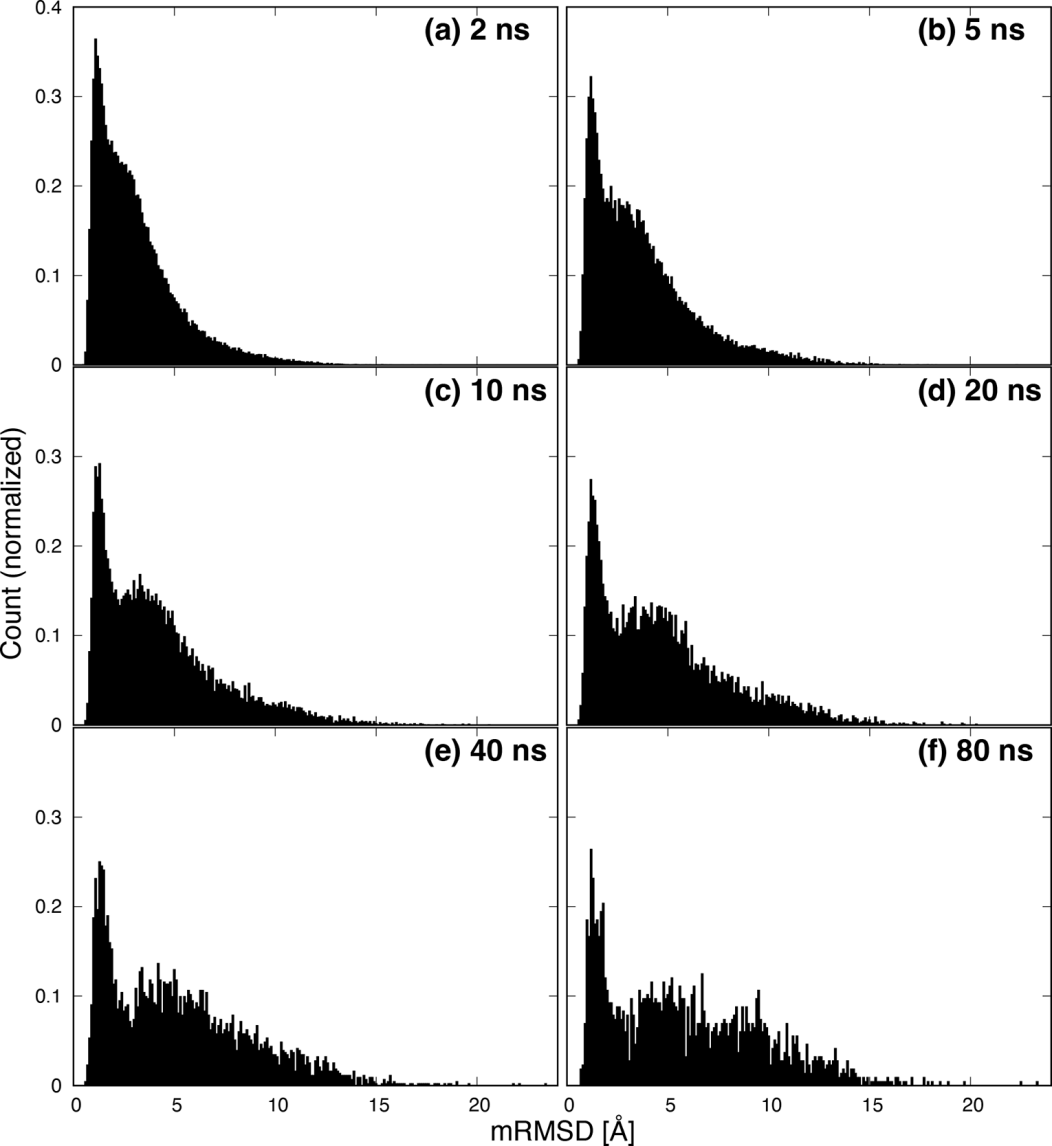
Histogram of mRMSD values. Panels a–f have time intervals Δ*t* of 2, 5, 10, 20, and 80 ns, respectively. Counts are normalized.

Figure 12 shows the histograms of mRMSD values for each time interval. No separation is observed in the peaks at (a) 2 ns or (b) 5 ns. At time intervals of ≥10 ns, separation of the peaks is observed. The splitting of the first peak observed in Trp-cage is not observed at (d) 20 ns. The separation of the first peak is a Trp-cage specific phenomenon. This is because NuG2 is larger and thus mRMSD values are less sensitive to terminal fluctuations. At (f) 80 ns, three peaks related to NuG2 dynamics were observed.

**Figure 13:**
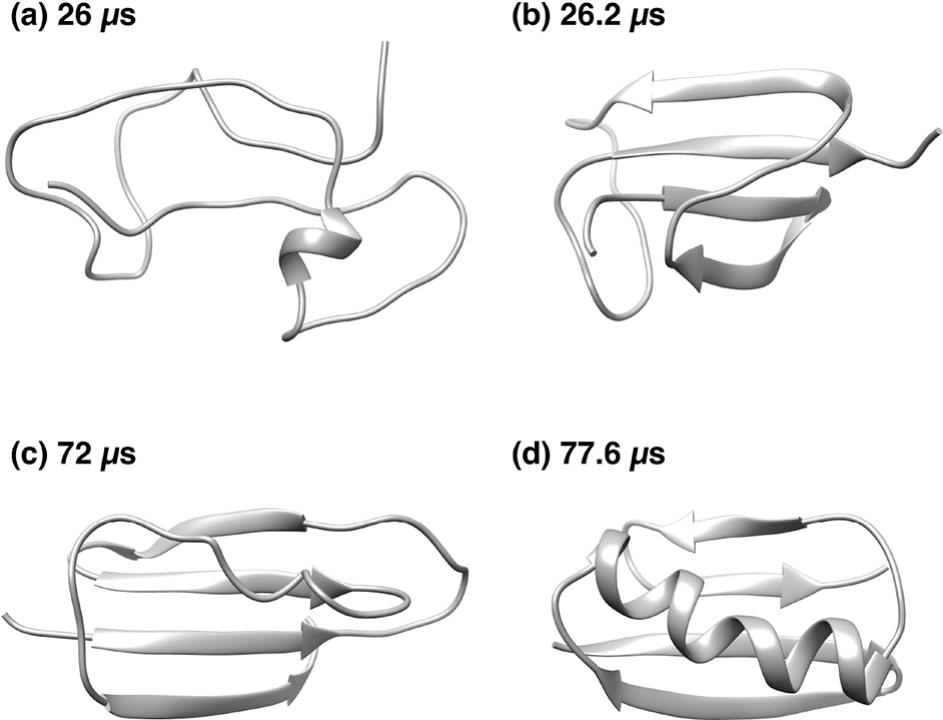
Backbone structures of NuG2 protein. (a) Random coil structure at beginning of the metastable region α in Figure 10c. (b) The metastable structure in the metastable region α. (c) The metastable structure in the metastable region β. (d) The most stable structure in the stable region A.

**Figure 14:**
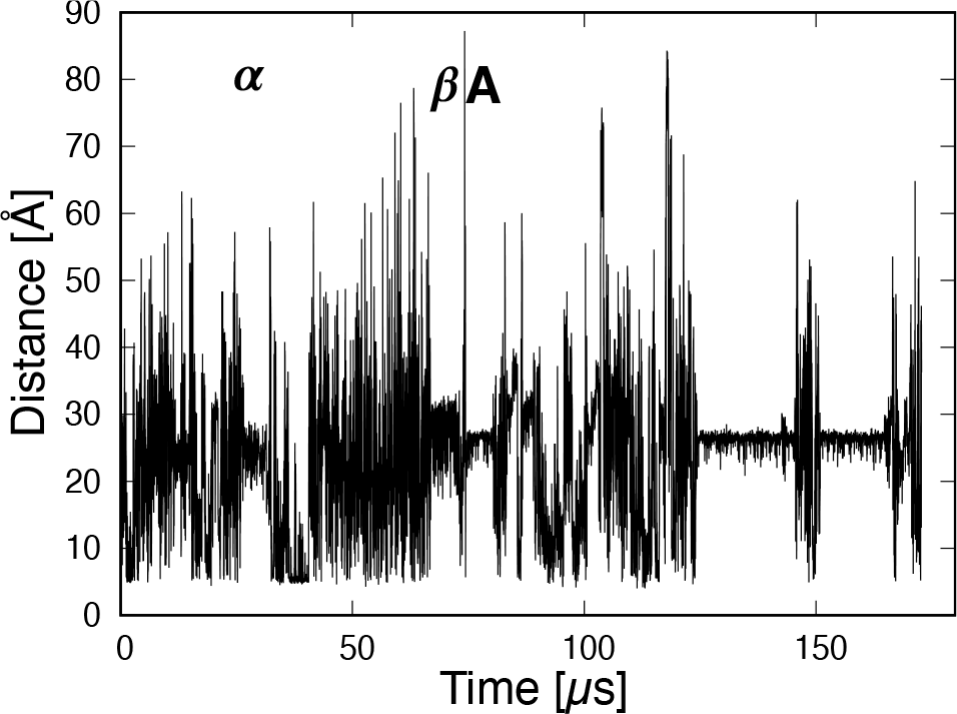
Time series of the end-to-end distance between the N- and C-termini. α, β and A are the same as in Figure 10c.

Figure 13 shows the characteristic structures of (a, b) the metastable state region α, (c) the metastable region β, and (d) the stable state region A. (a) Just before the start of region α, NuG2 forms a random coil structure. Immediately after, a parallel β-sheet is formed at the N- and C-termini. (b) This parallel β-sheet is stable, and then antiparallel β-sheets are formed between β_1_ and β_2_ and between β_3_ and β_4_. This structure resembles the native structure without the α-helix. This structure fits into B1 in the ref (22) classification. However, there is no direct transition from this metastable structure to the native structure. At the end of region α, the parallel β-sheet of β_1_ and β_2_ breaks down and NuG2 becomes a random coil structure. (c) In the β region, the structure is formed as shown in Figure 13c. This is classified as the R4 in ref (22). The structure which forms the parallel β-sheets between β_1_ and β_4_ strands and between β_2_ and β_3_ strands cannot be the expected structure. This structure also does not directly change to the native structure. Between regions β and A, the parallel β-sheets of β_1_ and β_4_ are broken and NuG2 protein takes on an extended structure. Between regions β and A, the end-to-end distance exceeds 80 Å, as observed in Figure 14. (d) Then, β_1_ and β_2_ strands form the antiparallel β-sheet and NuG2 folds rapidly to reach the native structure, as shown in Figure 13d.

The time series of the end-to-end distance between the N- and C-termini is shown in Figure 14. α, β, and A indicate the same regions as in Figure 10. Similar to RMSD and mRMSD, in addition to region A, regions of stable states starting from 125 and 155 μs can be observed. However, the value of the end-to-end distance of the native structure and the width of the fluctuation of the end-to-end distance depends on the type of protein.^44^ Therefore, it is not easy to search for stable states using only this measure. On the other hand, we can obtain information from the end-to-end distance in a different way from RMSD or mRMSD. For example, large values are observed at the end of the stable or metastable states. These indicate that the parallel β-sheet between β_1_ and β_4_ was broken. RMSD and mRMSD can only show that the structure changed significantly.

## Conclusions

We investigated protein folding processes by calculating the mRMSD, which does not require a reference structure. We have discussed here how the mRMSD can be incorporated into the stable and metastable states of proteins. In MD simulation trajectories of Trp-cage and NuG2 proteins, the time series of mRMSD can be used to correctly detect native structures. In addition, it is also able to detect the metastable states of both proteins. We also showed the difference in RMSD values when using each of the most stable structures within the MD simulation and the experimental structure as a reference. The misalignment between the reference structure and the most stable structure in the MD simulation affects the resolution of the energy analysis.

The mRMSD can be used to analyze MD simulations of proteins whose native structure is unknown. The behavior of the time series of the mRMSD can be examined to determine whether the simulation should be stopped. It is possible to determine with equal or higher accuracy than generating references with AlphaFold2,^99^ RoseTTAFold,^100^ etc. The flow of analyzing an MD simulation of a protein of unknown structure is as follows: 1. Perform the MD simulation, calculate mRMSD on the fly, and stop the simulation at the end of the stable state region after the stable state one appears several times. 2. Sample the structures of the stable state regions and determine the most stable structure by calculating the total energy or the frequency distribution. 3. Calculate conventional RMSD using the most stable structure as a reference, and analyze the various energies of the protein.

In general, it is difficult to extract native or metastable states from trajectories of MD simulations with large structural changes. The regions extracted by mRMSD calculation can be analyzed by other analytical methods to clarify the mechanisms of protein stability, dynamics, and folding processes. For example, the stability of the most stable and metastable states can be investigated by calculating total energy. The folding process can also be studied by analyzing the structures before and after the regions using clustering methods.

If a target is an intrinsically disordered protein (IDP) with partially flexible regions, this method may not work well. However, by splitting the structure into two or three parts, we can examine the behavior of RMSD of an IDP. An IDP undergoes transitions to more ordered states upon binding to its targets.^101^ Calculating mRMSD is also useful to investigate such binding processes between an IDP and its diverse partners.

The appropriate time interval is ≥20 ns in the case of Trp-cage and NuG2. The appropriate time interval depends on the system. For larger proteins, the appropriate time interval for mRMSD calculations would be longer. If the sampling interval Δ*s* and the mRMSD time interval Δ*t* are the same, the number of samples is smaller. However, the sampling interval and mRMSD time interval need not be the same. We can increase the number of samples by adjusting the time interval of the number of samples and the mRMSD time interval separately. We intend to make such improvement in future work.

## Data Availability

MD trajectory data was provided by D. E. Shaw RESEARCH (e-mail: inquiries@deshaw.com). 3D-RISM input/output, mRMSD code, and parameter files obtained from the present calculations can be downloaded from the following repository: https://github.com/omron-sinicx/mRMSD-paper-data. The code for mRMSD was written using MDAnalysis (ver. 2.3.0).^102^ The 3D-RISM with RMDFT functional code for the single GPU system is available from the following repository: https://github.com/drmaruyama/3D-RISM-CUDA/tree/rmdft.

## Supporting Information Available

The stable structures of Trp-cage and the time series of mRMSD of NuG2 (PDF)

## References

[ref1] Götz A. W.; Williamson M. J.; Xu D.; Poole D.; Le Grand S.; Walker R. C. Routine Microsecond Molecular Dynamics Simulations with AMBER on GPUs. 1. Generalized Born. J. Chem. Theory Comput. 2012, 8, 1542–1555. 10.1021/ct200909j. 22582031 PMC3348677

[ref2] Salomon-Ferrer R.; Götz A. W.; Poole D.; Le Grand S.; Walker R. C. Routine Microsecond Molecular Dynamics Simulations with AMBER on GPUs. 2. Explicit Solvent Particle Mesh Ewald. J. Chem. Theory Comput. 2013, 9, 3878–3888. 10.1021/ct400314y. 26592383

[ref3] Stone J. E.; Phillips J. C.; Freddolino P. L.; Hardy D. J.; Trabuco L. G.; Schulten K. Accelerating molecular modeling applications with graphics processors. J. Comput. Chem. 2007, 28, 2618–2640. 10.1002/jcc.20829. 17894371

[ref4] Phillips J. C.; Hardy D. J.; Maia J. D. C.; Stone J. E.; Ribeiro J. V.; Bernardi R. C.; Buch R.; Fiorin G.; Hénin J.; Jiang W.; McGreevy R.; Melo M. C. R.; Radak B. K.; Skeel R. D.; Singharoy A.; Wang Y.; Roux B.; Aksimentiev A.; Luthey-Schulten Z.; Kalé L. V.; Schulten K.; Chipot C.; Tajkhorshid E. Scalable molecular dynamics on CPU and GPU architectures with NAMD. J. Chem. Phys. 2020, 153, 044130 10.1063/5.0014475. 32752662 PMC7395834

[ref5] Eastman P.; Pande V. In GPU Computing Gems Jade ed.; Mei W., Hwu W., Eds.; Applications of GPU Computing Series; Morgan Kaufmann: Boston, 2012; pp 399–407.

[ref6] Abraham M. J.; Murtola T.; Schulz R.; Páll S.; Smith J. C.; Hess B.; Lindahl E. GROMACS: High performance molecular simulations through multi-level parallelism from laptops to supercomputers. SoftwareX 2015, 1–2, 19–25. 10.1016/j.softx.2015.06.001.

[ref7] Kutzner C.; Páll S.; Fechner M.; Esztermann A.; de Groot B. L.; Grubmüller H. Best bang for your buck: GPU nodes for GROMACS biomolecular simulations. J. Comput. Chem. 2015, 36, 1990–2008. 10.1002/jcc.24030. 26238484 PMC5042102

[ref8] Harvey M. J.; Giupponi G.; Fabritiis G. D. ACEMD: Accelerating Biomolecular Dynamics in the Microsecond Time Scale. J. Chem. Theory Comput. 2009, 5, 1632–1639. 10.1021/ct9000685. 26609855

[ref9] Narumi T.; Ohno Y.; Okimoto N.; T K.; Suenaga A.; Futatsugi N.; Yanai R.; Himeno R.; Fujikawa S.; Ikei M.; Taiji M. A 55 TFLOPS simulation of amyloid-forming peptides from yeast prion Sup35 with the special purpose computer system MDGRAPE-3. Proceedings of the SC06 (High Performance Computing, Networking, Storage and Analysis), Tampa, FL, USA 2006, 49 10.1145/1188455.1188506.

[ref10] Ohmura I.; Morimoto G.; Ohno Y.; Hasegawa A.; Taiji M. MDGRAPE-4: a special-purpose computer system for molecular dynamics simulations. Philos. Trans. R. Soc. A 2014, 372, 20130387 10.1098/rsta.2013.0387. PMC4084528 24982255

[ref11] Shaw D. E.; Deneroff M. M.; Dror R. O.; Kuskin J. S.; Larson R. H.; Salmon J. K.; Young C.; Batson B.; Bowers K. J.; Chao J. C.; Eastwood M. P.; Gagliardo J.; Grossman J. P.; Ho C. R.; Ierardi D. J.; Kolossváry I.; Klepeis J. L.; Layman T.; McLeavey C.; Moraes M. A.; Mueller R.; Priest E. C.; Shan Y.; Spengler J.; Theobald M.; Towles B.; Wang S. C. Anton, a Special-purpose Machine for Molecular Dynamics Simulation. Commun. ACM 2008, 51, 91–97. 10.1145/1364782.1364802.

[ref12] Shaw D. E.; Grossman J. P.; Bank J. A.; Batson B.; Butts J. A.; Chao J. C.; Deneroff M. M.; Dror R. O.; Even A.; Fenton C. H.; Forte A.; Gagliardo J.; Gill G.; Greskamp B.; Ho C. R.; Ierardi D. J.; Iserovich L.; Kuskin J. S.; Larson R. H.; Layman T.; Lee L.-S.; Lerer A. K.; Li C.; Killebrew D.; Mackenzie K. M.; Mok S. Y.-H.; Moraes M. A.; Mueller R.; Nociolo L. J.; Peticolas J. L.; Quan T.; Ramot D.; Salmon J. K.; Scarpazza D. P.; Ben Schafer U.; Siddique N.; Snyder C. W.; Spengler J.; Tang P. T. P.; Theobald M.; Toma H.; Towles B.; Vitale B.; Wang S. C.; Young C. Anton 2: Raising the Bar for Performance and Programmability in a Special-purpose Molecular Dynamics Supercomputer. Proc. Int. Conf. for High Performance Computing, Networking, Storage and Analysis. 2014, 41–53. 10.1109/SC.2014.9.

[ref13] Shaw D. E.; Adams P. J.; Azaria A.; Bank J. A.; Batson B.; Bell A.; Bergdorf M.; Bhatt J.; Butts J. A.; Correia T.; Dirks R. M.; Dror R. O.; Eastwood M. P.; Edwards B.; Even A.; Feldmann P.; Fenn M.; Fenton C. H.; Forte A.; Gagliardo J.; Gill G.; Gorlatova M.; Greskamp B.; Grossman J.; Gullingsrud J.; Harper A.; Hasenplaugh W.; Heily M.; Heshmat B. C.; Hunt J.; Ierardi D. J.; Iserovich L.; Jackson B. L.; Johnson N. P.; Kirk M. M.; Klepeis J. L.; Kuskin J. S.; Mackenzie K. M.; Mader R. J.; McGowen R.; McLaughlin A.; Moraes M. A.; Nasr M. H.; Nociolo L. J.; O’Donnell L.; Parker A.; Peticolas J. L.; Pocina G.; Predescu C.; Quan T.; Salmon J. K.; Schwink C.; Shim K. S.; Siddique N.; Spengler J.; Szalay T.; Tabladillo R.; Tartler R.; Taube A. G.; Theobald M.; Towles B.; Vick W.; Wang S. C.; Wazlowski M.; Weingarten M. J.; Williams J. M.; Yuh K. A. Anton 3: Twenty Microseconds of Molecular Dynamics Simulation before Lunch. Proceedings of the International Conference for High Performance Computing, Networking, Storage and Analysis. New York, NY, USA 2021, 1–11.

[ref14] Lindorff-Larsen K.; Piana S.; Dror R. O.; Shaw D. E. How fast-folding proteins fold. Science 2011, 334, 517–520. 10.1126/science.1208351. 22034434

[ref15] Mitsutake A.; Iijima H.; Takano H. Relaxation mode analysis of a peptide system: Comparison with principal component analysis. J. Chem. Phys. 2011, 135, 164102 10.1063/1.3652959. 22047223

[ref16] Nagai T.; Mitsutake A.; Takano H. Principal component relaxation mode analysis of an all-atom molecular dynamics simulation of human lysozyme. J. Phys. Soc. Jpn. 2013, 82, 023803 10.7566/JPSJ.82.023803.

[ref17] Mitsutake A.; Takano H. Relaxation mode analysis and Markov state relaxation mode analysis for chignolin in aqueous solution near a transition temperature. J. Chem. Phys. 2015, 143, 124111 10.1063/1.4931813. 26429000

[ref18] Karasawa N.; Mitsutake A.; Takano H. Two-step relaxation mode analysis with multiple evolution times applied to all-atom molecular dynamics protein simulation. Phys. Rev. E 2017, 96, 062408 10.1103/PhysRevE.96.062408. 29347325

[ref19] Karasawa N.; Mitsutake A.; Takano H. Improved Relaxation Mode Analysis of a Hen Egg-White Lysozyme Protein. Biophys. J. 2017, 112, 353a 10.1016/j.bpj.2016.11.1915.

[ref20] Mitsutake A.; Takano H. Relaxation mode analysis for molecular dynamics simulations of proteins. Biophys. Rev. 2018, 10, 375 10.1007/s12551-018-0406-7. 29546562 PMC5899748

[ref21] Karasawa N.; Mitsutake A.; Takano H. Identification of slow relaxation modes in a protein trimer via positive definite relaxation mode analysis. J. Chem. Phys. 2019, 150, 084113 10.1063/1.5083891. 30823754

[ref22] Mitsutake A.; Takano H. Folding pathways of NuG2–a designed mutant of protein G–using relaxation mode analysis. J. Chem. Phys. 2019, 151, 044117 10.1063/1.5097708. 31370539

[ref23] Maruyama Y.; Takano H.; Mitsutake A. Analysis of molecular dynamics simulations of 10-residue peptide, chignolin, using statistical mechanics: Relaxation mode analysis and three-dimensional reference interaction site model theory. Biophys. Physicobiol. 2019, 16, 407–429. 10.2142/biophysico.16.0_407. 31984194 PMC6975981

[ref24] Miyakawa T.; Sugimori K.; Kawaguchi K.; Takasu M.; Nagao H.; Morikawa R. Relationship between Dynamics of Structures and Dynamics of Hydrogen Bonds in Hras-GTP/GDP Complex. Proceedings of the 2020 10th International Conference on Bioscience, Biochemistry and Bioinformatics. New York, NY, USA, 2020, 1–7. 10.1145/3386052.3386059.

[ref25] Mori T.; Saito S. Dynamic heterogeneity in the folding/unfolding transitions of FiP35. J. Chem. Phys. 2015, 142, 135101 10.1063/1.4916641. 25854260

[ref26] Mori T.; Saito S. Molecular Mechanism Behind the Fast Folding/Unfolding Transitions of Villin Headpiece Subdomain: Hierarchy and Heterogeneity. J. Phys. Chem. B 2016, 120, 11683–11691. 10.1021/acs.jpcb.6b08066. 27769115

[ref27] Naritomi Y.; Fuchigami S. Slow dynamics in protein fluctuations revealed by time-structure based independent component analysis: The case of domain motions. J. Chem. Phys. 2011, 134, 065101 10.1063/1.3554380. 21322734

[ref28] Naritomi Y.; Fuchigami S. Slow dynamics of a protein backbone in molecular dynamics simulation revealed by time-structure based independent component analysis. J. Chem. Phys. 2013, 139, 215102 10.1063/1.4834695. 24320404

[ref29] Pérez-Hernández G.; Paul F.; Giorgino T.; De Fabritiis G.; Noé F. Identification of slow molecular order parameters for Markov model construction. J. Chem. Phys. 2013, 139, 015102 10.1063/1.4811489. 23822324

[ref30] Schwantes C. R.; Pande V. S. Improvements in Markov State Model Construction Reveal Many Non-Native Interactions in the Folding of NTL9. J. Chem. Theory Comput. 2013, 9, 2000–2009. 10.1021/ct300878a. 23750122 PMC3673732

[ref31] Schwantes C. R.; Pande V. S. Modeling Molecular Kinetics with tICA and the Kernel Trick. J. Chem. Theory Comput. 2015, 11, 600–608. 10.1021/ct5007357. 26528090 PMC4610300

[ref32] Takano H.; Miyashita S. Relaxation modes in random spin systems. J. Phys. Soc. Jpn. 1995, 64, 3688–3698. 10.1143/JPSJ.64.3688.

[ref33] Koseki S.; Hirao H.; Takano H. Monte Carlo Study of Relaxation Modes of a Single Polymer Chain. J. Phys. Soc. Jpn. 1997, 66, 1631–1637. 10.1143/JPSJ.66.1631.

[ref34] Hirao H.; Koseki S.; Takano H. Molecular dynamics study of relaxation modes of a single polymer chain. J. Phys. Soc. Jpn. 1997, 66, 3399–3405. 10.1143/JPSJ.66.3399.

[ref35] Kufareva I., Abagyan R. Homology modeling; Springer, 2011; pp 231–257.

[ref36] Carugo O. Statistical validation of the root-mean-square-distance, a measure of protein structural proximity. Protein Eng. Des. Sel. 2007, 20, 33–37. 10.1093/protein/gzl051. 17218333

[ref37] Harano Y.; Roth R.; Sugita Y.; Ikeguchi M.; Kinoshita M. Physical basis for characterizing native structures of proteins. Chem. Phys. Lett. 2007, 437, 112–116. 10.1016/j.cplett.2007.01.087.

[ref38] Kinoshita M. Importance of translational entropy of water in biological self-assembly processes like protein folding. Int. J. Mol. Sci. 2009, 10, 1064–1080. 10.3390/ijms10031064. 19399238 PMC2672019

[ref39] Oshima H.; Kinoshita M. A highly efficient hybrid method for calculating the hydration free energy of a protein. J. Comput. Chem. 2016, 37, 712–723. 10.1002/jcc.24253. 26576506

[ref40] Kajiwara Y.; Yasuda S.; Takamuku Y.; Murata T.; Kinoshita M. Identification of thermostabilizing mutations for a membrane protein w hose three-dimensional structure is unknown. J. Comput. Chem. 2017, 38, 211–223. 10.1002/jcc.24673. 27862099

[ref41] Maruyama Y.; Mitsutake A. Stability of unfolded and folded protein structures using a 3D-RISM with the RMDFT. J. Phys. Chem. B 2017, 121, 9881–9885. 10.1021/acs.jpcb.7b08487. 28969418

[ref42] Maruyama Y.; Mitsutake A. Analysis of Structural Stability of Chignolin. J. Phys. Chem. B 2018, 122, 3801–3814. 10.1021/acs.jpcb.8b00288. 29526100

[ref43] Maruyama Y.; Koroku S.; Imai M.; Takeuchi K.; Mitsutake A. Mutation-induced change in chignolin stability from π-turn to α-turn. RSC Adv. 2020, 10, 22797–22808. 10.1039/D0RA01148G. 35514567 PMC9054626

[ref44] Maruyama Y.; Mitsutake A. Structural Stability Analysis of Proteins Using End-to-End Distance: A 3D-RISM Approach. J 2022, 5, 114–125. 10.3390/j5010009.

[ref45] Harada R.; Sladek V.; Shigeta Y. Nontargeted Parallel Cascade Selection Molecular Dynamics Based on a Nonredundant Selection Rule for Initial Structures Enhances Conformational Sampling of Proteins. J. Chem. Inf. Model. 2019, 59, 5198–5206. 10.1021/acs.jcim.9b00753. 31697897

[ref46] Harada R.; Sladek V.; Shigeta Y. Nontargeted Parallel Cascade Selection Molecular Dynamics Using Time-Localized Prediction of Conformational Transitions in Protein Dynamics. J. Chem. Theory Comput. 2019, 15, 5144–5153. 10.1021/acs.jctc.9b00489. 31411882

[ref47] Yasuda T.; Morita R.; Shigeta Y.; Harada R. Independent Nontargeted Parallel Cascade Selection Molecular Dynamics (Ino-PaCS-MD) to Enhance the Conformational Sampling of Proteins. J. Chem. Theory Comput. 2021, 17, 5933–5943. 10.1021/acs.jctc.1c00558. 34410106

[ref48] Neidigh J. W.; Fesinmeyer R. M.; Andersen N. H. Designing a 20-residue protein. Nat. Struct. Biol. 2002, 9, 425–430. 10.1038/nsb798. 11979279

[ref49] Barua B.; Lin J. C.; Williams V. D.; Kummler P.; Neidigh J. W.; Andersen N. H. The Trp-cage: optimizing the stability of a globular miniprotein. Protein Eng. Des. Sel. 2008, 21, 171–185. 10.1093/protein/gzm082. 18203802 PMC3166533

[ref50] Nauli S.; Kuhlman B.; Baker D. Computer-based redesign of a protein folding pathway. Nat. Struct. Biol. 2001, 8, 602 10.1038/89638. 11427890

[ref51] Beglov D.; Roux B. An integral equation to describe the solvation of polar molecules in l iquid water. J. Phys. Chem. B 1997, 101, 7821–7826. 10.1021/jp971083h.

[ref52] Kovalenko A.; Hirata F. Three-dimensional density profiles of water in contact with a solute o f arbitrary shape: A RISM approach. Chem. Phys. Lett. 1998, 290, 237–244. 10.1016/S0009-2614(98)00471-0.

[ref53] Matubayasi N.; Nakahara M. Theory of solutions in the energetic representation. I. Formulation. J. Chem. Phys. 2000, 113, 6070–6081. 10.1063/1.1309013.

[ref54] Matubayasi N.; Nakahara M. Theory of solutions in the energy representation. II. Functional for the chemical potential. J. Chem. Phys. 2002, 117, 3605–3616. 10.1063/1.1495850.

[ref55] Matubayasi N.; Nakahara M. Theory of solutions in the energy representation. III. Treatment of the molecular flexibility. J. Chem. Phys. 2003, 119, 9686–9702. 10.1063/1.1613938.

[ref56] Nguyen C. N.; Kurtzman Young T.; Gilson M. K. Grid inhomogeneous solvation theory: Hydration structure and thermodynamics of the miniature receptor cucurbit[7]uril. J. Chem. Phys. 2012, 137, 044101 10.1063/1.4733951. 22852591 PMC3416872

[ref57] Nguyen C. N.; Young T. K.; Gilson M. K. Erratum: “Grid inhomogeneous solvation theory: Hydration structure and thermodynamics of the miniature receptor cucurbit[7]uril” [J. Chem. Phys. 137, 044101 (2012)]. J. Chem. Phys. 2012, 137, 149901 10.1063/1.4751113. PMC3416872 22852591

[ref58] Maruyama Y.; Harano Y. Does water drive protein folding?. Chem. Phys. Lett. 2013, 581, 85–90. 10.1016/j.cplett.2013.07.006.

[ref59] Pronk S.; Páll S.; Schulz R.; Larsson P.; Bjelkmar P.; Apostolov R.; Shirts M. R.; Smith J. C.; Kasson P. M.; Van Der Spoel D.; Hess B.; Lindahl E. GROMACS 4.5: A high-throughput and highly parallel open source molecular simulation toolkit. Bioinformatics 2013, 29, 845–854. 10.1093/bioinformatics/btt055. 23407358 PMC3605599

[ref60] MacKerell A. D.; Bashford D.; Bellott M.; Dunbrack R. L.; Evanseck J. D.; Field M. J.; Fischer S.; Gao J.; Guo H.; Ha S.; Joseph-McCarthy D.; Kuchnir L.; Kuczera K.; Lau F. T.; Mattos C.; Michnick S.; Ngo T.; Nguyen D. T.; Prodhom B.; Reiher W. E.; Roux B.; Schlenkrich M.; Smith J. C.; Stote R.; Straub J.; Watanabe M.; Wiórkiewicz-Kuczera J.; Yin D.; Karplus M. All-atom empirical potential for molecular modeling and dynamics studies of proteins. J. Phys. Chem. B 1998, 102, 3586–3616. 10.1021/jp973084f. 24889800

[ref61] Mackerell A. D.; Feig M.; Brooks C. L. Extending the treatment of backbone energetics in protein force fields: Limitations of gas-phase quantum mechanics in reproducing protein conformational distributions in molecular dynamics simulation. J. Comput. Chem. 2004, 25, 1400–1415. 10.1002/jcc.20065. 15185334

[ref62] Piana S.; Lindorff-Larsen K.; Shaw D. E. How robust are protein folding simulations with respect to force field parameterization?. Biophys. J. 2011, 100, L47–L49. 10.1016/j.bpj.2011.03.051. 21539772 PMC3149239

[ref63] Sumi T.; Mitsutake A.; Maruyama Y. A solvation-free-energy functional: A reference-modified density functional formulation. J. Comput. Chem. 2015, 36, 1359–1369. 10.1002/jcc.23942. 26032201

[ref64] Sumi T.; Mitsutake A.; Maruyama Y. Erratum: “A solvation-free-energy functional: A reference-modified density functional formulation” [J. Comput. Chem. 2015, 36, 1359–1369]. J. Comput. Chem. 2015, 2009 10.1002/jcc.24035. 26032201

[ref65] Maruyama Y. Correction terms for the solvation free energy functional of three-dimensional reference interaction site model based on the reference-modified density functional theory. J. Mol. Liq. 2019, 291, 111160 10.1016/j.molliq.2019.111160.

[ref66] Maruyama Y.; Hirata F. Modified Anderson method for accelerating 3D-RISM calculations using graphics processing unit. J. Chem. Theory Comput. 2012, 8, 3015–3021. 10.1021/ct300355r. 26605714

[ref67] Nukada A.; Maruyama Y.; Matsuoka S. High performance 3-D FFT using multiple CUDA GPUs. Proceedings of the 5th Annual Workshop on General Purpose Processing with Graphics Processing Units - GPGPU-5 2012, 57–63. 10.1145/2159430.2159437.

[ref68] Piana S.; Laio A. A Bias-Exchange Approach to Protein Folding. J. Phys. Chem. B 2007, 111, 4553–4559. 10.1021/jp067873l. 17419610

[ref69] Doshi U.; Hamelberg D. Achieving Rigorous Accelerated Conformational Sampling in Explicit Solvent. J. Phys. Chem. Lett. 2014, 5, 1217–1224. 10.1021/jz500179a. 26274474

[ref70] Zaborowski B.; Jagieła D.; Czaplewski C.; Hałabis A.; Lewandowska A.; Żmudzińska W.; Ołdziej S.; Karczyńska A.; Omieczynski C.; Wirecki T.; Liwo A. A Maximum-Likelihood Approach to Force-Field Calibration. J. Chem. Inf. Model. 2015, 55, 2050–2070. 10.1021/acs.jcim.5b00395. 26263302

[ref71] Miao Y.; Feixas F.; Eun C.; McCammon J. A. Accelerated molecular dynamics simulations of protein folding. J. Comput. Chem. 2015, 36, 1536–1549. 10.1002/jcc.23964. 26096263 PMC4487363

[ref72] Kamiya M.; Sugita Y. Flexible selection of the solute region in replica exchange with solute tempering: Application to protein-folding simulations. J. Chem. Phys. 2018, 149, 072304 10.1063/1.5016222. 30134668

[ref73] Harada R.; Shigeta Y. Temperature-Shuffled Structural Dissimilarity Sampling Based on a Root-Mean-Square Deviation. J. Chem. Inf. Model. 2018, 58, 1397–1405. 10.1021/acs.jcim.8b00095. 29882667

[ref74] Harada R.; Shigeta Y. Selection Rules for Outliers in Outlier Flooding Method Regulate Its Conformational Sampling Efficiency. J. Chem. Inf. Model. 2019, 59, 3919–3926. 10.1021/acs.jcim.9b00546. 31424213

[ref75] Omelyan I.; Kovalenko A. Enhanced solvation force extrapolation for speeding up molecular dynamics simulations of complex biochemical liquids. J. Chem. Phys. 2019, 151, 214102 10.1063/1.5126410. 31822083

[ref76] Simmerling C.; Strockbine B.; Roitberg A. E. All-Atom Structure Prediction and Folding Simulations of a Stable Protein. J. Am. Chem. Soc. 2002, 124, 11258–11259. 10.1021/ja0273851. 12236726

[ref77] Chowdhury S.; Lee M. C.; Xiong G.; Duan Y. Ab initio Folding Simulation of the Trp-cage Mini-protein Approaches NMR Resolution. J. Mol. Biol. 2003, 327, 711–717. 10.1016/S0022-2836(03)00177-3. 12634063

[ref78] Paschek D.; Hempel S.; García A. E. Computing the stability diagram of the Trp-cage miniprotein. Proc. Natl. Acad. Sci. U.S.A. 2008, 105, 17754–17759. 10.1073/pnas.0804775105. 19004791 PMC2582582

[ref79] Andryushchenko V. A.; Chekmarev S. F. A hydrodynamic view of the first-passage folding of Trp-cage miniprotein. Eur. Biophys J. 2016, 45, 229–243. 10.1007/s00249-015-1089-7. 26559408

[ref80] Yasuda T.; Shigeta Y.; Harada R. The Folding of Trp-cage is Regulated by Stochastic Flip of the Side Chain of Tryptophan. Chem. Lett. 2021, 50, 162–165. 10.1246/cl.200699.

[ref81] Zhou R. Trp-cage: Folding free energy landscape in explicit water. Proc. Natl. Acad. Sci. U.S.A. 2003, 100, 13280–13285. 10.1073/pnas.2233312100. 14581616 PMC263783

[ref82] Ding F.; Buldyrev S. V.; Dokholyan N. V. Folding Trp-Cage to NMR Resolution Native Structure Using a Coarse-Grained Protein Model. Biophys. J. 2005, 88, 147–155. 10.1529/biophysj.104.046375. 15533926 PMC1304993

[ref83] Iavarone A. T.; Patriksson A.; van der Spoel D.; Parks J. H. Fluorescence Probe of Trp-Cage Protein Conformation in Solution and in Gas Phase. J. Am. Chem. Soc. 2007, 129, 6726–6735. 10.1021/ja065092s. 17487969

[ref84] Patriksson A.; Adams C. M.; Kjeldsen F.; Zubarev R. A.; van der Spoel D. A Direct Comparison of Protein Structure in the Gas and Solution Phase: The Trp-cage. J. Phys. Chem. B 2007, 111, 13147–13150. 10.1021/jp709901t. 17973523

[ref85] Scian M.; Lin J. C.; Le Trong I.; Makhatadze G. I.; Stenkamp R. E.; Andersen N. H. Crystal and NMR structures of a Trp-cage mini-protein benchmark for computational fold prediction. Proc. Natl. Acad. Sci. U.S.A. 2012, 109, 12521–12525. 10.1073/pnas.1121421109. 22802678 PMC3411959

[ref86] Kannan S.; Zacharias M. Role of Tryptophan Side Chain Dynamics on the Trp-Cage Mini-Protein Folding Studied by Molecular Dynamics Simulations. PLoS One 2014, 9, e88383 10.1371/journal.pone.0088383. 24563686 PMC3921895

[ref87] Byrne A.; Williams D. V.; Barua B.; Hagen S. J.; Kier B. L.; Andersen N. H. Folding Dynamics and Pathways of the Trp-Cage Miniproteins. Biochemistry 2014, 53, 6011–6021. 10.1021/bi501021r. 25184759 PMC4179588

[ref88] Marinelli F.; Pietrucci F.; Laio A.; Piana S. A Kinetic Model of Trp-Cage Folding from Multiple Biased Molecular Dynamics Simulations. PLoS Comput. PLoS Comput. Biol. 2009, 5, e1000452 10.1371/journal.pcbi.1000452. 19662155 PMC2711228

[ref89] Marino K. A.; Bolhuis P. G. Confinement-Induced States in the Folding Landscape of the Trp-cage Miniprotein. J. Phys. Chem. B 2012, 116, 11872–11880. 10.1021/jp306727r. 22954175

[ref90] Han W.; Schulten K. Characterization of Folding Mechanisms of Trp-Cage and WW-Domain by Network Analysis of Simulations with a Hybrid-Resolution Model. J. Phys. Chem. B 2013, 117, 13367–13377. 10.1021/jp404331d. 23915394 PMC3811923

[ref91] Beauchamp K. A.; McGibbon R.; Lin Y.-S.; Pande V. S. Simple few-state models reveal hidden complexity in protein folding. Proc. Natl. Acad. Sci. U. S. A. 2012, 109, 17807–17813. 10.1073/pnas.1201810109. 22778442 PMC3497769

[ref92] Best R. B.; Hummer G.; Eaton W. A. Native contacts determine protein folding mechanisms in atomistic simulations. Proc. Natl. Acad. Sci. U. S. A. 2013, 110, 17874–17879. 10.1073/pnas.1311599110. 24128758 PMC3816414

[ref93] Zheng W.; Best R. B. Reduction of All-Atom Protein Folding Dynamics to One-Dimensional Diffusion. J. Phys. Chem. B 2015, 119, 15247–15255. 10.1021/acs.jpcb.5b09741. 26601695 PMC6197821

[ref94] Schwantes C. R.; Shukla D.; Pande V. S. Markov State Models and tICA Reveal a Nonnative Folding Nucleus in Simulations of NuG2. Biophys. J. 2016, 110, 1716–1719. 10.1016/j.bpj.2016.03.026. 27119632 PMC4850345

[ref95] Becerra D.; Butyaev A.; Waldispühl J. Fast and flexible coarse-grained prediction of protein folding routes using ensemble modeling and evolutionary sequence variation. Bioinformatics 2019, 36, 1420–1428. 10.1093/bioinformatics/btz743. 31584628

[ref96] Shao Q.; Zhu W. Nonnative contact effects in protein folding. Phys. Chem. Chem. Phys. 2019, 21, 11924–11936. 10.1039/C8CP07524G. 31134232

[ref97] Chang L.; Perez A.; Miranda-Quintana R. A. Improving the analysis of biological ensembles through extended similarity measures. Phys. Chem. Chem. Phys. 2021, 24, 444–451. 10.1039/D1CP04019G. 34897334

[ref98] Chang L.; Perez A. Deciphering the Folding Mechanism of Proteins G and L and Their Mutants. J. Am. Chem. Soc. 2022, 144, 14668–14677. 10.1021/jacs.2c04488. 35930769

[ref99] Jumper J.; Evans R.; Pritzel A.; Green T.; Figurnov M.; Ronneberger O.; Tunyasuvunakool K.; Bates R.; Žídek A.; Potapenko A.; Bridgland A.; Meyer C.; Kohl S. A. A.; Ballard A. J.; Cowie A.; Romera-Paredes B.; Nikolov S.; Jain R.; Adler J.; Back T.; Petersen S.; Reiman D.; Clancy E.; Zielinski M.; Steinegger M.; Pacholska M.; Berghammer T.; Bodenstein S.; Silver D.; Vinyals O.; Senior A. W.; Kavukcuoglu K.; Kohli P.; Hassabis D. Highly accurate protein structure prediction with AlphaFold. Nature 2021, 596, 583–589. 10.1038/s41586-021-03819-2. 34265844 PMC8371605

[ref100] Baek M.; DiMaio F.; Anishchenko I.; Dauparas J.; Ovchinnikov S.; Lee G. R.; Wang J.; Cong Q.; Kinch L. N.; Schaeffer R. D.; Millán C.; Park H.; Adams C.; Glassman C. R.; DeGiovanni A.; Pereira J. H.; Rodrigues A. V.; van Dijk A. A.; Ebrecht A. C.; Opperman D. J.; Sagmeister T.; Buhlheller C.; Pavkov-Keller T.; Rathinaswamy M. K.; Dalwadi U.; Yip C. K.; Burke J. E.; Garcia K. C.; Grishin N. V.; Adams P. D.; Read R. J.; Baker D. Accurate prediction of protein structures and interactions using a three-track neural network. Science 2021, 373, 871–876. 10.1126/science.abj8754. 34282049 PMC7612213

[ref101] Mohan A.; Oldfield C. J.; Radivojac P.; Vacic V.; Cortese M. S.; Dunker A. K.; Uversky V. N. Analysis of Molecular Recognition Features (MoRFs). J. Mol. Biol. 2006, 362, 1043–1059. 10.1016/j.jmb.2006.07.087. 16935303

[ref102] Michaud-Agrawal N.; Denning E. J.; Woolf T. B.; Beckstein O. MDAnalysis: A toolkit for the analysis of molecular dynamics simulations. J. Comput. Chem. 2011, 32, 2319–2327. 10.1002/jcc.21787. 21500218 PMC3144279

[ref103] Pettersen E. F.; Goddard T. D.; Huang C. C.; Couch G. S.; Greenblatt D. M.; Meng E. C.; Ferrin T. E. UCSF Chimera—A visualization system for exploratory research and analysis. J. Comput. Chem. 2004, 25, 1605–1612. 10.1002/jcc.20084. 15264254

