# Supplementary material for: Analysis of Protein Folding Simulation with Moving Root Mean Square Deviation
Source: J Chem Inf Model. 2023 Feb 23;63(5):1529–41. doi: 10.1021/acs.jcim.2c01444 (PMC10015464; doi:10.1021/acs.jcim.2c01444)
Supplement: Supplementary file 1 — ci2c01444_si_001.pdf [file ci2c01444_si_001.pdf]

Supporting Information: Analysis of Protein Folding Simulation with Moving Root Mean Square Deviation

Yutaka Maruyama,[*,†,‡] Ryo Igarashi,[†] Yoshitaka Ushiku,[†] and Ayori Mitsutake[‡]

†*OMRON SINIC X Corporation, Tokyo 113-0033, Japan*

‡*Department of Physics, School of Science and Technology, Meiji University, 1-1-1 Higashi-Mita, Tama-ku, Kawasaki-shi, Kanagawa 214-8571, Japan*

* E-mail: yutaka.maruyama@sinicx.com

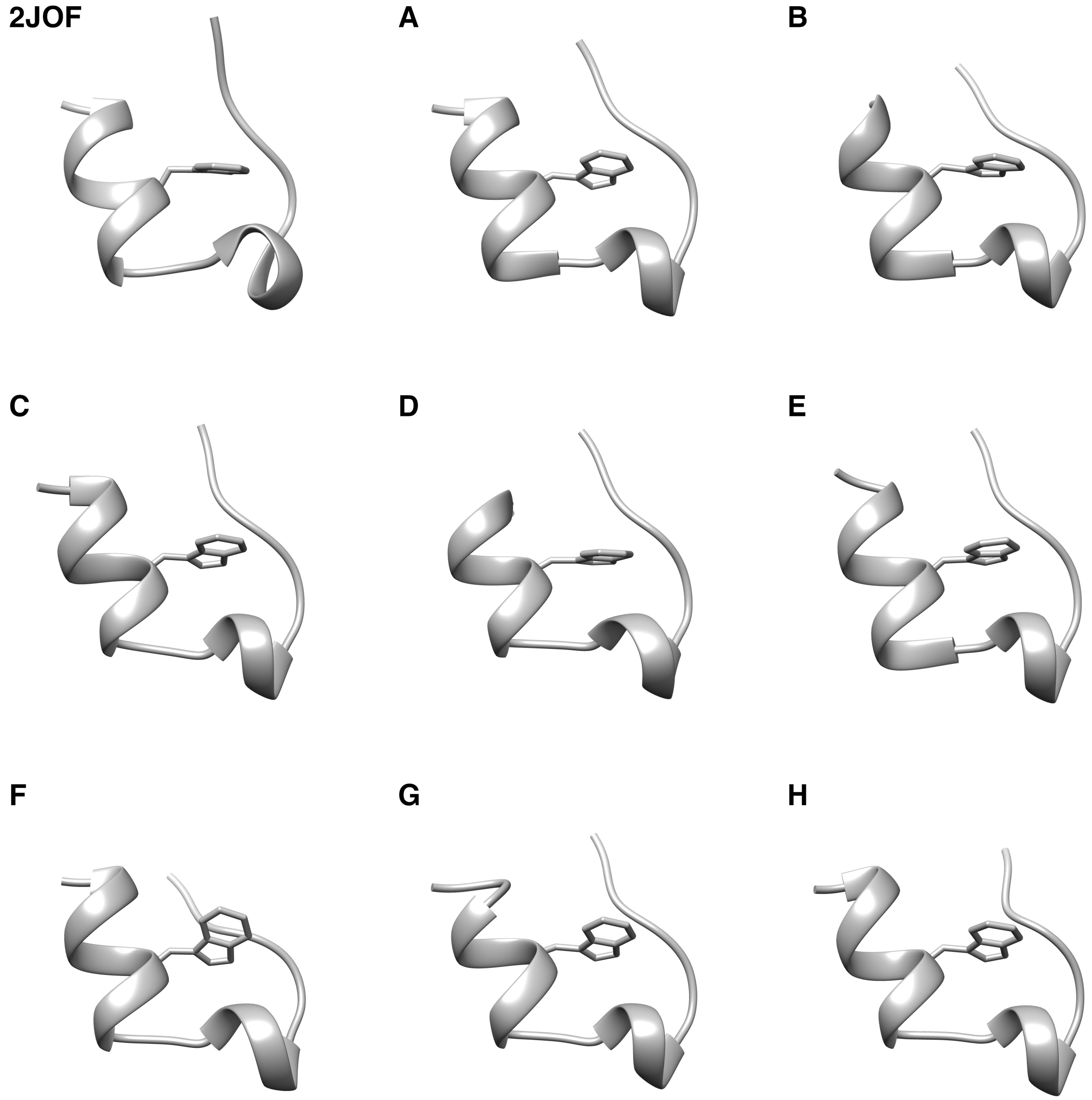

Figure S1 (Supporting Information)

Backbone structures with the side chain of 6th amino acid tryptophan. (Upper left) PDB 2JOF model 1. (Other) A to H correspond to the stable state regions A-H in Figure 1.

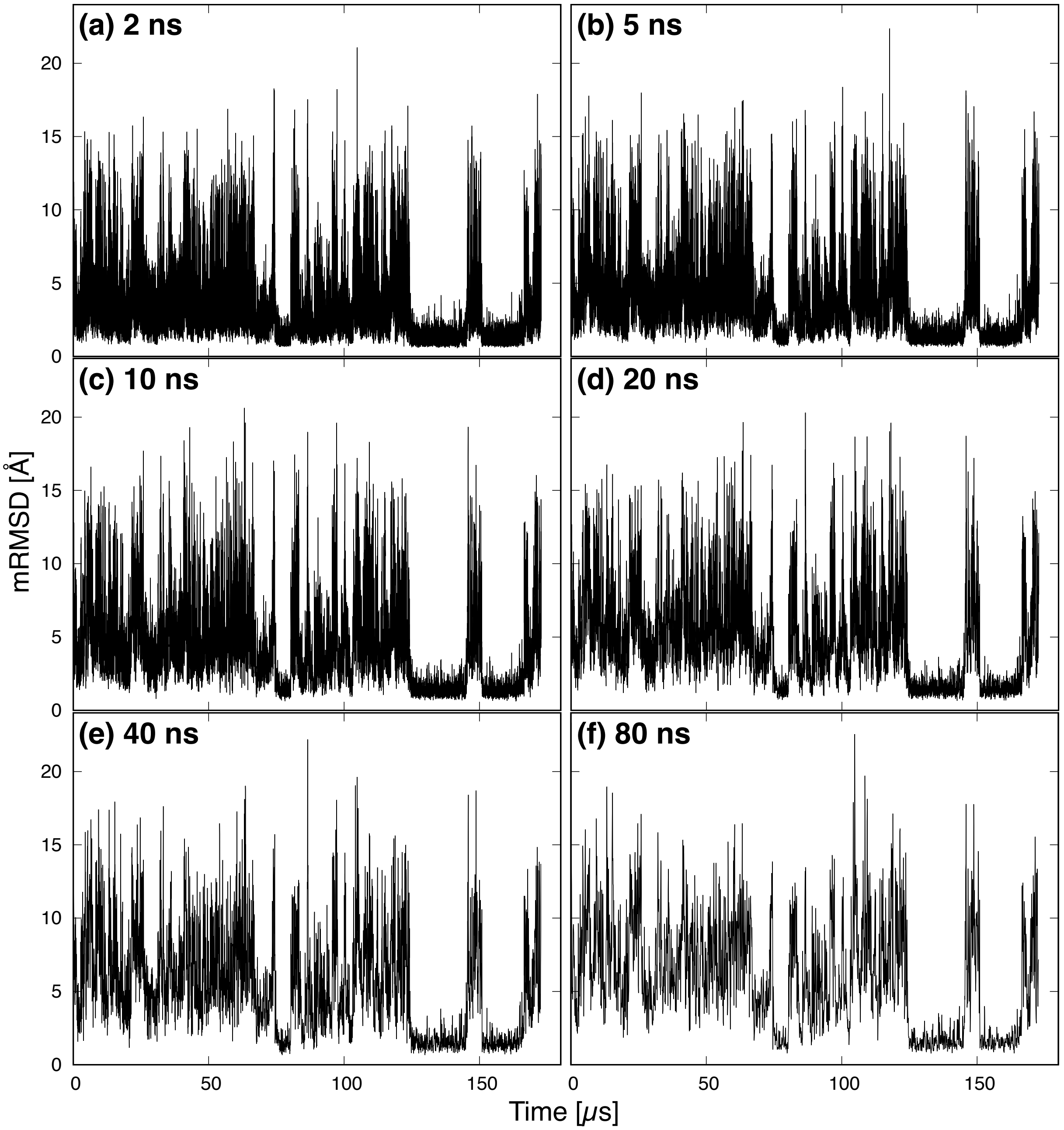

Figure S2 (Supporting Information)

Time series of the moving root-mean-square deviation (mRMSD) of NuG2 protein. (a)-(f) have time intervals Δt of 2, 5, 10, 20, 40, and 80 ns, respectively.
